# Anti-BCMA/CD19 CAR T cell therapy in patients with refractory generalized myasthenia gravis: a single-arm, phase 1 trial

**DOI:** 10.1016/j.eclinm.2025.103621

**Published:** 2025-10-31

**Authors:** Yong Zhang, Dan Liu, Zhouao Zhang, Xiaoyu Huang, Jiang Cao, Gang Wang, Huizhong Li, Shengli Li, Shenyang Zhang, Wei Zhang, Hao Chen, Xue Du, Zhouyi Wang, Mingjin Yang, Tiancheng Luo, Xinyan Guo, Tianyu Ma, Deyou Peng, Guoyan Qi, Shenghua Zong, Guiyun Cui, Junnian Zheng, Ming Shi

**Affiliations:** a Department of Neurology, The Affiliated Hospital of Xuzhou Medical University, Xuzhou, Jiangsu, China; b Cancer Institute, Cellular Therapeutics School of Medicine, Xuzhou Medical University, Xuzhou, Jiangsu, China; c Jiangsu Center for the Collaboration and Innovation of Cancer Biotherapy, Xuzhou Medical University, Xuzhou, Jiangsu, China; d Center of Clinical Oncology, The Affiliated Hospital of Xuzhou Medical University, Xuzhou, Jiangsu, China; e Department of Hematology, The Affiliated Hospital of Xuzhou Medical University, Xuzhou, Jiangsu, China; f Clinical Research Institute, The Affiliated Hospital of Xuzhou Medical University, Xuzhou, Jiangsu, China; g Center of Treatment of Myasthenia Gravis, People's Hospital of Shijiazhuang, Shijiazhuang, Heibei, China; h KingMed Diagnostic Laboratory, Guangzhou, China

**Keywords:** Refractory myasthenia gravis, Chimeric antigen receptor T cell, Clinical trial, B-Cell maturation antigen, CD19

## Abstract

**Background:**

Treating refractory generalized myasthenia gravis is still a big challenge, so new treatments are needed. Several studies have shown the potential of chimeric antigen receptor (CAR) T cell therapy in the treatment of autoimmune diseases. However, no clinical trial of bispecific CAR T cells targeting refractory myasthenia gravis has been done. We developed autologous anti-B-cell maturation antigen (BCMA) and CD19 bispecific CAR T cells to evaluate the safety and efficacy in refractory myasthenia gravis.

**Methods:**

This single-arm, phase 1 trial (ChiCTR2200061267) was conducted at the Affiliated Hospital of Xuzhou Medical University in refractory generalized myasthenia gravis patients (Myasthenia Gravis Activities of Daily Living [MG-ADL] ≥6-point, and Quantitative Myasthenia Gravis scale [QMG] ≥8-point). Anti-BCMA/CD19 bispecific CAR T cells were administered at 1.0 × 10^6^, 3.0 × 10^6^, and 5.0 × 10^6^ CAR T cells per kg. Primary endpoints assessed safety (dose-limiting toxicities, maximum tolerated dose, and adverse events); secondary endpoints assessed disease severity and other related indexes.

**Findings:**

Between May 3, 2023, and June 19, 2024, 18 patients were enrolled and received apheresis and a single CAR T cell infusion. Mean age was 41 years (SD 12) and 12 (67%) participants were female. The anti-BCMA/CD19 bispecific CAR T cells were generally safe, with no dose-limiting toxicities, no immune effector cell-associated neurotoxicity syndrome, and only grade 1 cytokine release syndrome (39% of patients). The most common grade 3 or worse adverse events within 28 days were hematological toxicities, including three leukopenia, three neutropenia, one anaemia, and one thrombocytopenia. All were transient and manageable. 17 participants completed follow-up. Clinical improvements by day 180 were −8.6 (95% CI −10.2 to −7.0) for MG-ADL score and −15.4 (−17.6 to −13.2) for QMG score. 14 participants (82%) achieved minimal manifestations, 15 (88%) stopped glucocorticoids, all stopped non-steroidal immunosuppressants, and eight (47%) had anti-acetylcholine receptor antibody negative seroconversion.

**Interpretation:**

Anti-BCMA/CD19 CAR T cells showed good safety and efficacy in refractory generalized myasthenia gravis. A large proportion of participants had a minimal manifestation status and discontinued glucocorticoids and non-steroidal immunosuppressants, and a certain proportion of anti-acetylcholine receptor antibodies turned negative.

**Funding:**

10.13039/501100001809National Natural Science Foundation of China, Basic Research Program of Jiangsu, Young academic leaders of Jiangsu Qinglan Project, and Construction Project of High Level Hospital of Jiangsu Province.

**Research in context**

**Evidence before this study**

We searched the PubMed database on June 20, 2025, for relevant clinical studies on the use of chimeric antigen receptor (CAR) T cells therapy in myasthenia gravis, with no date or language restrictions. The search terms used were “chimeric antigen receptor” (or “CAR”) and “myasthenia gravis”. We found a multicentre, open-label, non-randomised phase 1b/2a study on autologous RNA CAR T cells and some case reports on single-target (CD19 or BCMA) CAR T cells in myasthenia gravis. However, there were no published clinical trial of bispecific CAR T cells for the treatment of refractory myasthenia gravis.

**Added value of this study**

To our knowledge, this was the first clinical trial evaluating anti-BCMA/CD19 bispecific CAR T cells in refractory myasthenia gravis. Our study showed that anti-BCMA/CD19 bispecific CAR T cells had good safety and efficacy in patients with refractory generalized myasthenia gravis. Treatment of participants with a single CAR T-cell infusion at three dose levels was associated with clinically meaningful improvements. These included the induction of a high proportion of minimal manifestations (14/17, 82%), the reduction of the glucocorticoid dose to ≤5 mg per day in all 17 participants, discontinuation of glucocorticoids (15 cases, 88%), and the negative seroconversion of the anti-acetylcholine receptor antibody in eight (47%) participants.

**Implications of all the available evidence**

Our data show that anti-BCMA/CD19 bispecific CAR T cells hold promise as a potential treatment option for refractory generalized myasthenia gravis and may provide a potential immune reset through a single infusion for myasthenia gravis. However, the long-term efficacy and safety of anti-BCMA/CD19 bispecific CAR T cells require further investigation in randomized, double-blind, placebo-controlled trials.

## Introduction

Myasthenia gravis is a prototypical autoimmune disorder characterized by pathogenic autoantibodies that target postsynaptic neuromuscular junction proteins.^1^ Despite rapid advances in immunotherapy, approximately 15–20% of patients develop refractory myasthenia gravis. These patients exhibit persistent symptoms despite an adequate dosage and duration of glucocorticoids combined with non-steroidal immunosuppressants, or they have intolerable side effects or require repeated intravenous immunoglobulin or plasma exchange.^2^ Given that the generation of pathogenic autoantibodies by B cells is the primary pathogenic mechanism of myasthenia gravis, B-cell depletion therapy is, in theory, an effective treatment strategy.^3^^,^^4^ Two randomized studies (BeatMG and RINOMAX) of rituximab, a chimeric anti-CD20 monoclonal antibody, did not achieve consistent positive results.^5^^,^^6^ This inconsistent result may be related to the fact that anti-CD20 monoclonal antibodies cannot eliminate mature plasma cells.^7^ The monoclonal antibody targeting CD19 has a broader spectrum and may, therefore, have better efficacy. The phase 3 trial of inebilizumab, a humanized anti-CD19 monoclonal antibody, achieved its primary endpoint with a significant reduction in Myasthenia Gravis Activities of Daily Living scale (MG-ADL).^8^ However, conventional B-cell-depleting monoclonal antibodies have limitations, such as incomplete B-cell depletion in deep tissues, transient effects, and repeated infusions. There is still a need for therapies inducing deep and sustained immune reconstitution.

Chimeric antigen receptor (CAR) T-cell therapy, initially developed for hematologic malignancies, emerged as a groundbreaking treatment paradigm for autoimmune diseases, probably achieving profound B-cell depletion and offering the potential for a single-infusion immune reset.^9^^,^^10^ Early reports on single-target CAR T cells in systemic lupus erythematosus, myasthenia gravis, and other autoimmune diseases provided the transformative potential. For instance, anti-CD19 CAR T cells induced sustained, drug-free remission in patients with systemic lupus erythematosus, accompanied by autoantibody normalization and achieved clinical improvement in a case of refractory myasthenia gravis.^9^^,^^11^ B-cell maturation antigen (BCMA) is another therapeutic target, highly expressed on long-lived bone marrow plasma cells that sustain antibody production. Anti-BCMA RNA CAR T cells demonstrated both efficacy and safety in myasthenia gravis, though repeated administrations were required.^12^ Another anti-BCMA CAR T cells reduced antibody levels in myasthenia gravis and suggested B-cell lineage reconstitution.^13^ However, two studies reported that one patient with chronic inflammatory demyelinating polyneuropathy experienced recurrence after anti-BCMA CAR T cells, and two patients with autoimmune hemolytic anemia, who had experienced recurrence after anti-CD19 CAR T cells, were successfully treated with a BCMA-Targeted T-Cell Engager.^14^^,^^15^ Thus, dual-targeting BCMA/CD19 CAR T-cells represent a strategic advancement, simultaneously eliminating B-cell from pro-B cells to long-lived plasma cells. This dual-targeting strategy was initially validated in multiple myeloma and later applied to systemic lupus erythematosus with a favorable safety profile.^16^^,^^17^

Despite these advances, no systematic clinical studies have evaluated dual-target CAR T cells in myasthenia gravis. Here, we report the phase 1 trial results of BCMA/CD19 bispecific CAR T cells in refractory generalized myasthenia gravis, assessing its safety and efficacy.

## Methods

### Study design and participants

This was a single-arm, phase 1 dose-escalation clinical trial evaluating the safety and efficacy of anti-BCMA/CD19 bispecific CAR T cells in patients with refractory generalized myasthenia gravis. We conducted the clinical trial at the Affiliated Hospital of Xuzhou Medical University, Xuzhou, Jiangsu, China.

Eligible participants were 14–70 years old, had a confirmed diagnosis of refractory myasthenia gravis (with a Myasthenia Gravis Foundation of America [MGFA] classification of Ⅱ, Ⅲ, or Ⅳ), had a score of Myasthenia Gravis Activities of Daily Living Scale (MG-ADL) of at least 6-point and a score of Quantitative Myasthenia Gravis scale (QMG) of at least 8-point, with more than 50% of the score attributed to non-ocular items at screening and before lymphodepleting chemotherapy, had positive tests for anti-acetylcholine receptor antibody, had been receiving a stable dose of glucocorticoids or non-steroidal immunosuppressants (or both), had an Eastern Cooperative Oncology Group (ECOG) performance status of 0 or 1, and had adequate organ function (alanine aminotransferase <3 times the upper limit of normal [ULN], aspartate aminotransferase <3 times ULN, total bilirubin <2.0 mg/dl, serum creatinine [Scr] <2.5 mg/dl). Participants also had appropriate peripheral venous access, had no contraindications for apheresis, and were able to receive chemotherapy. The major exclusion were the presence of any mental or psychological illnesses that could potentially interfere with the completion of treatment; active infections including human immunodeficiency virus, hepatitis B virus, hepatitis C virus, mycobacterium tuberculosis; highly allergic constitution or history of severe allergies, especially to interleukin (IL)-2; intravenous immunoglobulin, plasma exchange, neonatal Fc receptor inhibitors, or complement inhibitors within four weeks before lymphodepleting chemotherapy; thymectomy or B-cell clearance therapy within three months before lymphodepleting chemotherapy. The detailed inclusion and exclusion criteria are provided in the Supplementary Material 1.

### Ethics

The study was conducted in accordance with the principles of the Declaration of Helsinki. The study protocol, amendments, and informed consent were reviewed and approved by the Ethics Committee of the Affiliated Hospital of Xuzhou Medical University (number: XYFY2022-KL057-02). All participants provided written informed consent. This study was registered with the Chinese Clinical Trial Registry (ChiCTR2200061267) on June 18, 2022.

### Procedures

The CAR T cells were derived from the participant's autologous T cells infected with lentiviral vectors for expressing secondary generation CAR construction containing the bispecific tandem single-chain variable fragments, CD8 α Hinge and transmembrane domains, 4-1BB co-stimulatory domains, and CD3 ξ Activation domain. Details of the CAR T-cell construction process were provided in the Supplementary Material 1. Participants received lymphodepleting chemotherapy with fludarabine (30 mg/m^2^/day) and cyclophosphamide (300 mg/m^2^/day) for three consecutive days (days −5 to −3). During dose escalation, participants received a single infusion of anti-BCMA/CD19 bispecific CAR T cells at escalating doses: 1.0 × 10^6^/kg (dose level 1), 3.0 × 10^6^/kg (dose level 2), and 5.0 × 10^6^/kg (dose level 3) to establish the recommended dose for expansion.

Safety was assessed based on dose-limiting toxicities occurring within the first 28 days following CAR T-cell infusion. Adverse events were comprehensively documented. Participants were followed at months 3–6, 9, 12, and every 6 months thereafter until month 36, for a total of 3 years. The severity of adverse events was graded in accordance with the National Cancer Institute's Common Terminology Criteria for Adverse Events, version 5.0. Cytokine release syndrome and immune effector cell-associated neurotoxicity syndrome (ICANS) were defined and graded according to the criteria of the American Society for Transplantation and Cellular Therapy. Efficacy was assessed based on MG-ADL score, QMG score, and Myasthenia Gravis Quality of Life 15-revised (MG-QOL15r) score. QMG score was evaluated at least 8 h after the last use of cholinesterase inhibitor. The in-vivo amplification of anti-BCMA/CD19 CAR T cells in the peripheral blood of patients after infusion was evaluated by quantitative real-time polymerase chain reaction (qPCR).

Permissible concomitant medications for myasthenia gravis were pyridostigmine, glucocorticoids (maximum daily dose equivalent to 40 mg prednisone), and non-steroidal immunosuppressants (azathioprine, tacrolimus, mycophenolate mofetil, cyclophosphamide). Pyridostigmine dose had to be stable for at least one week, glucocorticoids for at least four weeks, and non-steroidal immunosuppressants for at least three months before lymphodepleting chemotherapy. Glucocorticoids and non-steroidal immunosuppressants doses were not permitted to increase and dose tapering could be decided at the discretion of the site investigator after CAR T-cell infusion.

All participants were closely monitored after lymphodepleting chemotherapy and CAR T-cell infusion. For neutrophil deficiency (absolute neutrophil count less than 1.0 × 10^9^/L), granulocyte colony-stimulating factor and prophylactic antibiotics (mostly sulfamethoxazole and cephalosporins) were given. Other antibiotic uses were based on infection signs, fever, or persistent neutrophil/white blood cell deficiency. For those with severe infections or hypogammaglobulinemia (IgG less than 4 g/L), 0.4–0.6 g/kg of intravenous immunoglobulin was administered. More detailed management approaches for adverse events were provided in Supplementary Material 1.

### Outcomes

The primary endpoint was the safety of anti-BCMA/CD19 bispecific CAR T cells, including dose-limiting toxicities and maximum tolerated dose, to set the recommended dose for the dose expansion and phase 2 trials. Dose-limiting toxicity was defined as any therapy-related grade 4 life-threatening toxicities, or grade 3 or worse cytokine release syndrome not improving to grade 2 or lower within 72 h, grade 4 hematological toxicities not improving to grade 3 or lower within 14 days, and grade 3 or worse non-hematological toxicities not improving to grade 2 or lower within 72 h. The secondary endpoints were the changes in MG-ADL score, QMG score, MG-QOL15r score from day 0 (baseline) at each follow-up assessment up to 36 months. Clinical meaningful improvement was defined as a 2-point reduction in MG-ADL score or a 3-point reduction in QMG score. Predefined exploratory endpoints included the proportion of participants achieving minimal manifestations or better based on MGFA Post-Intervention Status (MGFA-PIS), changes in anti-acetylcholine receptor antibody levels, and the proportion of those with glucocorticoid dosage tapered to 5 mg or less daily.

### Statistical analysis

This was an exploratory, single-arm study with a dose-escalation regimen, so all the analysis are descriptive in nature. The planned sample size was 18 patients and was calculated based on a design of three to six participants per group to determine the maximum tolerated dose in the dose escalation stage. As prespecified in the protocol, safety and efficacy analysis were done in all participants who received the anti-BCMA/CD19 bispecific CAR T cells. Statistical Package for Social Sciences 26.0 software for Windows (SPSS, Chicago, IL, USA) and GraphPad Prism 9.2.0 software (GraphPad Software, La Jolla, CA, USA) for statistical analysis. Categorical variables were expressed as numbers (percentages), normally distributed continuous variables were expressed as mean and standard deviation (SD), non-normally distributed variables continuous were expressed as median and interquartile range (IQR). Changes in MG-ADL, QMG, and MG-QOL15r scores of all participants who received the anti-BCMA/CD19 bispecific CAR T cells were expressed as mean change with a 95% confidence interval (CI). The cutoff date for all analysis was April 1, 2025.

### Role of the funding source

The funder of the study had no role in study design, data collection, data analysis, data interpretation, or writing of the report.

## Results

**Fig. 1:**
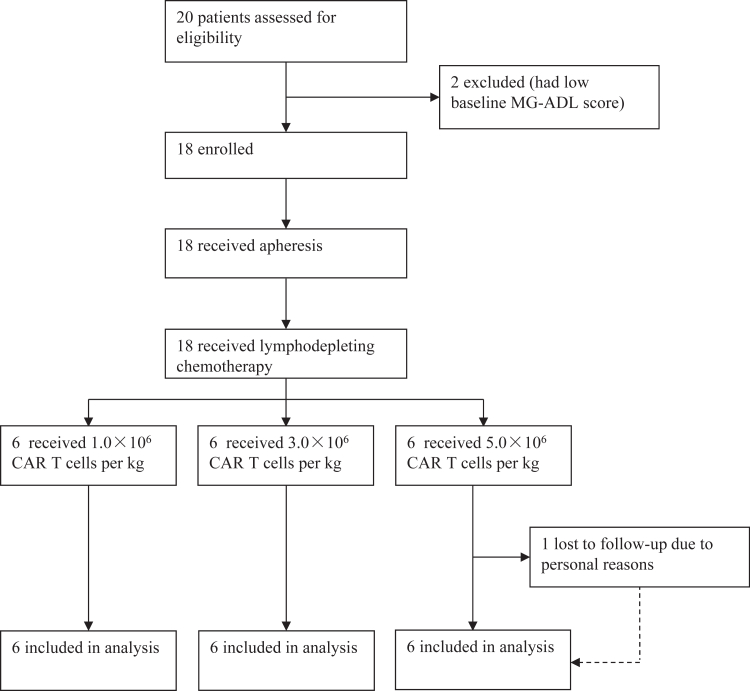
**Trial profile**. CAR = chimeric antigen receptor. MG-ADL = Myasthenia Gravis Activities of Daily Living. All 18 eligible participants were included in the primary analysis of the endpoint.

Between May 3, 2023, and June 19, 2024, we screened 20 participants with refractory generalized myasthenia gravis who were positive for anti-acetylcholine receptor antibody. Two participants were excluded due to a low MG-ADL score (n = 2), and 18 participants were enrolled and received apheresis. The manufacture of anti-BCMA/CD19 bispecific CAR T cells was successful for all participants (n = 18). All 18 participants received lymphodepleting chemotherapy, CAR T-cell infusion, and were evaluated for safety and efficacy (Fig. 1). One patient in dose level 3 lost to follow-up >28 days after CAR T-cell infusion for personal reasons. The mean age of the participants was 41 years (SD 12). Most participants were female (67%). Six (33%) had thymectomy, nine (50%) had myasthenic crisis. All participants had previously been treated with glucocorticoids combined with at least one non-steroidal immunosuppressants. Most participants previously received intravenous immunoglobulin (67%), four (22%) had plasma exchange, eight (44%) had neonatal Fc receptor inhibitor, and two (11%) had complement inhibitor. Most participants had MGFA class III (67%) (Table 1).

**Table 1:** Demographics and baseline characteristic.

	Dose level 1 (n = 6)	Dose level 2 (n = 6)	Dose level 3 (n = 6)	Total (n = 18)
Sex, No. (%)^a^				
Male	3 (50)	0	3 (50)	6 (33)
Female	3 (50)	6 (100)	3 (50)	12 (67)
Age, years	48 (12)	40 (9)	37 (12)	42 (12)
BMI, kg/m^2^	24.8 (3.1)	19.6 (2.9)	25.5 (4.6)	22.8 (3.8)
Race, No. (%)				
Chinese	6 (100)	6 (100)	6 (100)	18 (100)
Age at onset, years	42 (13)	30 (12)	25 (14)	33 (14)
Disease duration, months	69 (59)	118 (56)	141 (134)	83 (42–185)
Anti-AChR antibody titer, nmol/L	57.9 (57.1)	6.5 (1.0–170.8)	11.7 (4.8–116.8)	14.8 (4.9–102.5)
Baseline score				
MG-ADL	9.7 (3.8)	8.3 (2.7)	9.8 (4.3)	8.0 (6.0–12.2)
QMG	16.3 (6.2)	17.5 (3.8)	19.2 (4.3)	17.7 (4.7)
MG-QOL15r	20.3 (3.1)	19.3 (4.2)	18.7 (3.6)	19.4 (3.5)
MGFA classification, No. (%)				
II	1 (17)	0	0	1 (6)
III	4 (67)	4 (67)	4 (67)	12 (67)
IV	1 (17)	2 (33)	2 (33)	5 (28)
Thymoma, No. (%)	4 (67)	0	0	4 (22)
Thymectomy, No. (%)	4 (67)	1 (17)	1 (17)	6 (33)
Myasthenia crisis history, No. (%)	2 (33)	4 (67)	3 (50)	9 (50)
Previous use of immunosuppressive treatments, No. (%)				
≥3	3 (50)	3 (50)	5 (83)	11 (61)
≥2	6 (100)	6 (100)	6 (100)	18 (100)
Type of immunosuppressive treatments, No. (%)				
Glucocorticoid	6 (100)	6 (100)	6 (100)	18 (100)
Azathioprine	0 (0)	1 (17)	3 (50)	4 (22)
Mycophenolate mofetil	1 (17)	1 (17)	3 (50)	5 (28)
Tacrolimus	6 (100)	6 (100)	4 (67)	16 (89)
Cyclophosphamide	2 (33)	2 (33)	1 (17)	5 (28)
Previous use of biological agents, No. (%)				
Rituximab	2 (33)	1 (17)	2 (33)	5 (28)
Eculizumab	1 (17)	1 (17)	0 (0)	2 (11)
Efgartigimod	3 (50)	1 (17)	4 (67)	8 (44)
Previous use of intravenous immunoglobulin, No. (%)	5 (83)	5 (83)	2 (33)	12 (67)
Previous use of plasma exchange, No. (%)	2 (33)	0	2 (33)	4 (22)
Dose of glucocorticoids at baseline, mg/day	22.1 (10.5)	16.7 (9.3)	20.4 (7.8)	19.7 (9.1)
Immunosuppressive treatments at baseline, No. (%)				
Glucocorticoid only	1 (17)	2 (33)	2 (33)	5 (28)
NSISTs only	0	0	0	0
Glucocorticoid plus NSIST	5 (83)	4 (67)	3 (50)	12 (67)
Type of NSIST used at baseline, No. (%)				
Azathioprine	0	0	1 (17)	1 (6)
Mycophenolate mofetil	0	0	1 (17)	1 (6)
Tacrolimus	4 (67)	4 (67)	1 (17)	9 (50)
Cyclophosphamide	1 (17)	0	0	1 (6)

Data are mean (SD), n (%), or median (range). AChR = acetylcholine receptor. BMI = Body Mass Index. MG-ADL = Myasthenia Gravis Activities of Daily Living. MGFA = Myasthenia Gravis Foundation of America. MG-QOL15r = Myasthenia Gravis Quality of Life 15-revised. QMG = Quantitative Myasthenia Gravis. NSIST = non-steroidal immunosuppressant.

a Sex was self-reported by participants from the options of male or female.

**Fig. 2:**
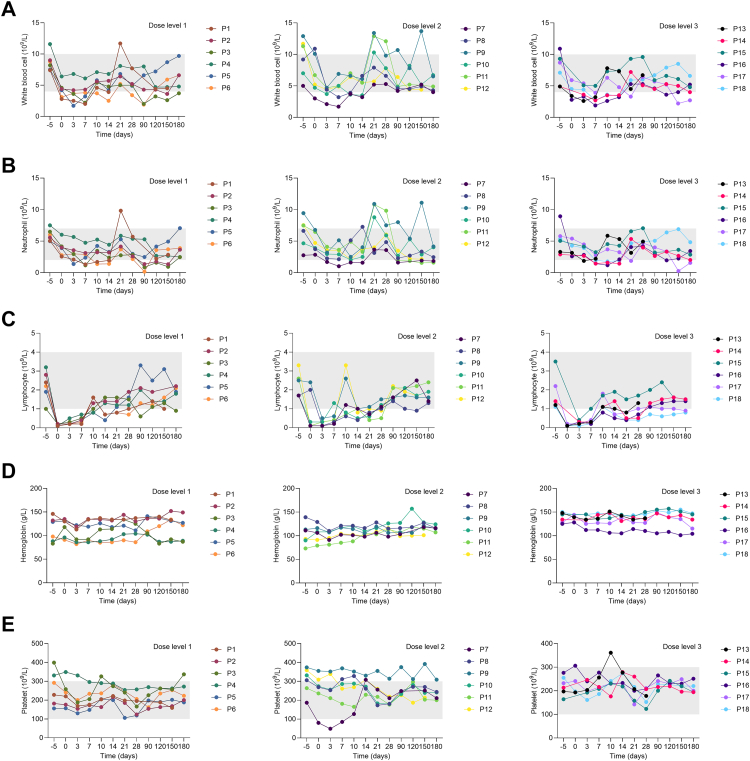
**Monitoring of hematological toxicity after lymphodepleting chemotherapy and CAR T-cell infusion**. Plots show change from day −5 to day 180 in observed count for white blood cell (A), neutrophil (B), lymphocyte (C), hemoglobin (D), and platelet (E). Participants in dose level 1 (n = 6), dose level 2 (n = 6), and dose level 3 (n = 6) received a dose of 1.0 × 10^6^, 3.0 × 10^6^, and 5.0 × 10^6^ anti-BCMA/CD19 CAR T cells per kg, respectively. Grey area indicates the reference range. CAR = chimeric antigen receptor.

No dose-limited toxicity was observed in all 18 participants. Anti-BCMA/CD19 bispecific CAR T cells had a tolerable safety profile at all doses (Table 2). No grade 5 adverse event occurred. The most common grade 3-4 adverse events within 28 days after infusion were hematological toxicities: three (17%) had leukopenia, 17 (94%) had lymphopenia, one (6%) had anemia, and one (6%) had thrombocytopenia. Two leukopenia cases occurred on day 7 and day 3, and all recovered in three days. The median duration of grade 3–4 lymphopenia was 10.0 days (IQR 7.0–10.0), the duration of grade 3 anemia was three days, and the duration of grade 3 thrombocytopenia was four days. Within 28 days, except for three (17%) patients who had grade 3 cough, no other grade 3 or worse non-hematological toxicity was observed. The most common grade 1–2 non-hematological toxicities were muscle soreness (16/18, 89%). The most common grade 3-4 adverse events >28 days after infusion were also hematological toxicities, including one (6%) patient had leukopenia and three (18%) patients had neutropenia. One patient had grade 3 leukopenia and grade 4 neutropenia on day 90 and quickly recovered after being treated with recombinant human granulocyte colony-stimulating factor. The other two cases of neutropenia occurred on day 90 and day 150, respectively. Since no definite infection occurred, no intervention was administrated, and both recovered at the next follow-up. The detailed changes in white blood cell, neutrophil, lymphocyte, hemoglobin, and platelet in three groups from day −5 to day 180 were shown in Fig. 2.

**Table 2:** Adverse events.

	Grade 1	Grade 2	Grade 3	Grade 4	Grade 1	Grade 2	Grade 3	Grade 4
	28 days post-CAR T-cell (n = 18)				>28 days post-CAR T-cell (n = 17)			
Cytokine release syndrome, No. (%)	7 (39)	0	0	0	0	0	0	0
Immune effector cell–associated neurotoxicity syndrome, No. (%)	0	0	0	0	0	0	0	0
Haematological, No. (%)								
Leukopenia	3 (17)	6 (33)	3 (17)	0	2 (12)	2 (12)	1 (6)	0
Neutropenia	3 (17)	7 (39)	0	0	4 (24)	1 (6)	1 (6)	2 (12)
Lymphopenia	0	1 (6)	6 (33)	11 (61)	2 (12)	2 (12)	0	0
Anaemia	2 (11)	6 (33)	1 (6)	0	6 (35)	2 (12)	0	0
Thrombocytopenia	0	0	1 (6)	0	0	0	0	0
Febrile neutropenia	0	0	0	0	0	0	0	0
Gastrointestinal disorders, No. (%)								
Diarrhoea	10 (56)	0	0	0	8 (47)	0	0	0
Abdominal pain	9 (50)	0	0	0	2 (12)	0	0	0
Nausea	5 (28)	0	0	0	1 (6)	0	0	0
Vomiting	2 (11)	0	0	0	0	0	0	0
Toothache	2 (11)	0	0	0	6 (35)	0	0	0
Metabolism and nutrition disorders, No. (%)								
Hypokalaemia	7 (39)	0	0	0	5 (29)	0	0	0
Hyponatraemia	1 (6)	0	0	0	0	0	0	0
Hypocalcaemia	2 (11)	1 (6)	0	0	9 (53)	0	0	0
Hypophosphataemia	3 (17)	0	0	0	0	0	0	0
Hypoalbuminaemia	9 (50)	0	0	0	11 (65)	0	0	0
Respiratory diseases, No. (%)								
Cough	4 (22)	8 (44)	3 (17)	0	7 (41)	6 (35)	0	0
Pneumonia	0	0	0	0	0	2 (12)	1 (6)	0
Other, No. (%)								
Fever	7 (39)	0	0	0	9 (53)	0	0	0
Conjunctivitis	5 (28)	0	0	0	6 (35)	0	0	0
Skin herpes	2 (12)	1 (6)	0	0	0	1 (6)	0	0
Increased alanine aminotransferase	1 (6)	0	0	0	0	0	0	0
Increased aspartate aminotransferase	0	0	0	0	0	0	0	0
Increased creatinine	0	0	0	0	0	0	0	0
Increased total bilirubin	0	0	0	0	0	0	0	0
Headache	9 (50)	1 (6)	0	0	2 (12)	0	0	0
Hand numbness	10 (56)	0	0	0	7 (41)	0	0	0
Muscle soreness	15 (83)	1 (6)	0	0	13 (76)	0	0	0
Chills	5 (28)	0	0	0	4 (24)	0	0	0
Night sweats	11 (61)	0	0	0	8 (47)	0	0	0

There were no deaths. Data are n (%).

Cytokine release syndrome occurred in seven (39%) participants with a median onset of 9.0 days (IQR 8.0–21.0) after CAR T-cell infusion and a duration from 1 to 3 days. All cytokine release syndrome were grade 1 and only one patient received dexamethasone to control. The median times for high-sensitivity C-reactive protein (hs-CRP) and IL-6 to reach their peak concentrations in participants with cytokine release syndrome were 17.0 days (IQR 10.0–21.0) and 10.0 days (IQR 7.0–21.0) after CAR T-cell infusion, respectively. No ICANS were observed in all participants by the cut-off date in this study.

Infections were observed in three (17%) patients, including one patient who had bacterial pneumonia and grade 2 viral infection, and two patients who had bacterial pneumonia. Bacterial pneumonia happened >28 days after CAR T-cell infusion, including one patient with grade 3 pneumonia and two participants with grade 2 pneumonia. A viral infection happened within 28 days after the CAR T-cell infusion.

**Fig. 3:**
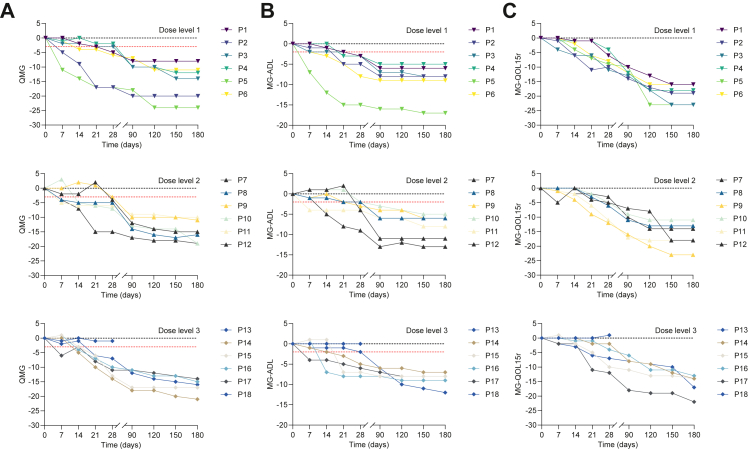
**Change from baseline in disease severity scores for participants**. Plots show change from day 0 to day 180 in observed scores for QMG (A), MG-ADL (B), and MG-QOL15r (C). Participants in dose level 1 (n = 6), dose level 2 (n = 6), and dose level 3 (n = 6) received a dose of 1.0 × 10^6^, 3.0 × 10^6^, and 5.0 × 10^6^ anti-BCMA/CD19 CAR T cells per kg, respectively. The dashed red lines indicate clinical meaningful improvements in MG-ADL (a reduction of ≥2-point) and QMG scores (a reduction of ≥3-point). MG-ADL = Myasthenia Gravis Activities of Daily Living. QMG = Quantitative Myasthenia Gravis. MG-QOL15r = Myasthenia Gravis Quality of Life 15-revised.

**Fig. 4:**
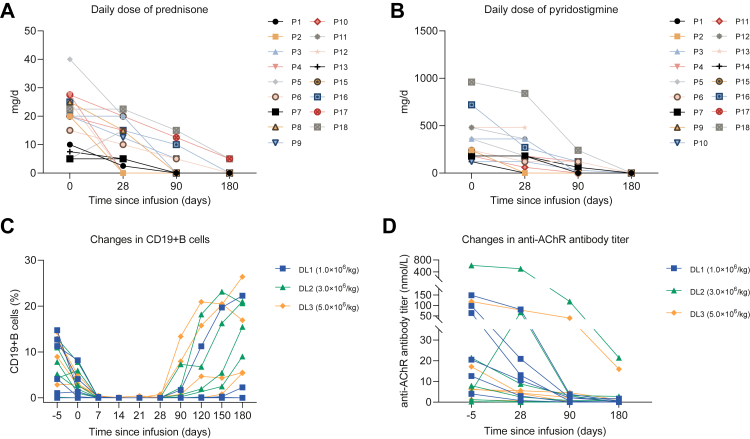
**Correlated drugs tapering, B-cell depletion, and anti-AChR antibody reduction following CAR T-cell therapy**. Plots show change in daily dose of prednisone (A) and pyridostigmine (B), the proportion of circulating CD19+ B cells among total lymphocytes (C), and anti-AChR antibody titer (D) after CAR T-cell infusion. AChR = acetylcholine receptors. CAR = chimeric antigen receptor. DL = dose level.

Although patient 13 was lost to follow-up, the other 17 participants showed reductions in MG-ADL, QMG, and MG-QOL15r scores from day 28 to day 180 (Fig. 3). The mean changes in MG-ADL, QMG, and MG-QOL15r scores of those 17 participants from day 0 to day 180 were −8.6 (95% CI −10.2 to −7.0), −15.4 (95% CI −17.6 to −13.2), and −17.2 (95% CI, −19.1 to −15.2), respectively. On day 180, all 17 participants (100%) had a ≥2-point improvement in MG-ADL score and a ≥3-point improvement in QMG score without disease exacerbation, 15 of 17 participants (88%) had a ≥6-point improvement in MG-ADL score, and all 17 (100%) participants had a ≥8-point improvement in QMG score on day 180 (Table 3). The proportion of participants who achieved status of minimal manifestations was 14 of 17 (82%) on day 180 (Table 3). Before the CAR T-cell infusion, 17 of 18 participants (94%) received glucocorticoid with a daily dose of 19.7 (9.1) mg equivalent to prednisone (Fig. 4A). On day 180, of the 17 participants who completed the follow-up, 15 participants (88%) discontinued their glucocorticoid, and two participants tapered their dose of prednisone from 27.5 mg daily and 22.5 mg daily to 5 mg daily (Fig. 4A and Table 3). 17 participants received pyridostigmine with a daily dose of 240 (IQR 180–420) mg before CAR T-cell infusion, and all 16 participants who completed follow-up discontinued on day 180 (Fig. 4B). 12 participants took non-steroidal immunosuppressants before the infusion, and all discontinued them within 180 days after the infusion.

**Table 3:** Measures of disease severity on day 180.

	All patients who completed follow-up (n = 17)	Dose level 1 (n = 6)	Dose level 2 (n = 6)	Dose level 3 (n = 5)
Mean score changes (95% CI)				
MG-ADL	−8.6 (−10.2 to −7.0)	−8.8 (−13.3 to −4.4)	−8.2 (−11.5 to −4.8)	−8.8 (−11.2 to −6.4)
QMG	−15.4 (−17.6 to −13.2)	−14.8 (−21.1 to −8.5)	−15.0 (−19.0 to −11.0)	−16.6 (−20.0 to −13.2)
MG-QOL15r	−17.2 (−19.1 to −15.2)	−19.2 (−20.7 to −15.8)	−16.2 (−20.7 to −11.6)	−16.0 (−20.7 to −11.4)
Number of participants with improvement, No. (%)				
MG-ADL decrease ≥2 points	17 (100)	6 (100)	6 (100)	6 (100)
QMG decrease ≥3 points	17 (100)	6 (100)	6 (100)	6 (100)
MG-ADL decrease ≥6 points	15 (88)	6 (100)	5 (83)	4 (80)
QMG decrease ≥8 points	17 (100)	6 (100)	6 (100)	6 (100)
Minimal manifestations	14 (82)	5 (83)	5 (83)	4 (80)
Prednisone ≤5 mg per day	17 (100)	6 (100)	6 (100)	6 (100)
Withdraw prednisone	15 (88)	6 (100)	6 (100)	3 (60)

Data are mean (95% CI) and n (%). MG-ADL = Myasthenia Gravis Activities of Daily Living. MG-QoL-15r = Myasthenia Gravis Quality of Life 15-revised. QMG = Quantitative Myasthenia Gravis.

The changes in circulating CD19+ B cells were detected by flow cytometry and serum concentrations of IgG, IgM, and IgA were detected by immunonephelometric (Fig. 4C and Supplementary Material 2 p 13). Deficiency of CD19+ B cells already existed in three (17%) participants before chemotherapy due to prior rituximab therapy (>3 months before CAR-T infusion). Another 15 participants developed B-cell aplasia after CAR T-cell infusion within 28 days. The clearance of circulating CD19+ B cells in most participants occurred on day 7 (11 of 18, 61%) and day 14 (2 of 18, 11%) after CAR T-cell infusion (Fig. 4C). Up to day 180, ten (56%) participants had B-cell reconstitution, in which B-cell reconstitution was first observed on day 90 in five (28%) participants (Fig. 4C). The median time of B-cell reconstitution in those participants was 105 (IQR 90–158) days after CAR T-cell infusion. Hypogammaglobulinemia (serum IgG <4 g/L) occurred in 15 (83%) participants, with a median onset time of 90 (IQR 21–90) days after infusion. Among those participants, only three participants’ IgG recovered within 180 days after infusion. IgM deficiency (defined as <0.5 g/L) already existed in four (22.2%) participants before CAR T-cell infusion and became more serious after infusion. Continuous decrease of IgM was observed in all participants, and none of them recovered within 180 days. IgA deficiency (defined as <1.0 g/L) already existed in four (22.2%) participants before infusion and occurred in all participants after CAR T-cell infusion. Only one participant recovered within 180 days.

All 18 enrolled participants tested positive for anti-acetylcholine receptor antibody at screening. The median antibody titer tested on day −5 before chemotherapy was 14.8 (IQR 4.9–102.5) nmol/L (tested by radioimmunoassay) (Fig. 4D). Patient 8 exhibited a titer of 0.26 nmol/L on day −5, which fell below the reference threshold of 0.4 nmol/L. The titers of acetylcholine receptor antibody were dynamically tested in 17 participants. The median titers were 12.6 (IQR 4.6–80.8) nmol/L on day −5 and decreased to 5.5 (IQR 1.6–44.0) nmol/L on day 28 (Fig. 4D). On day 90, the anti-acetylcholine receptor antibody turned negative in five participants and increased to eight on day 180 (Fig. 4D).

The in vivo expansion of CAR-T cells was detected in peripheral blood using qPCR within 28 days after CAR-T cell infusion (Supplementary Material 2 p 14). Amplification of CAR T cells peaked on day 14 in 16 (89%) participants and peaked on day 21 in two (11%) participants. The time appearance of amplification peak and the peck copies of CAR T cells had no association with the dose of CAR T-cell infusion. The amplification of CAR T cells rapidly decreased 28 days after CAR T-cell infusion.

## Discussion

In this single-arm trial of 18 patients with refractory generalized myasthenia gravis, anti-BCMA/CD19 bispecific CAR T cells were safe and associated with significant clinical improvement. These improvements were sustained in 17 participants who completed follow-up in all dose levels within 180 days. In addition, the status of minimal manifestations was achieved in 14 participants and 15 participants discontinued glucocorticoids 180 days after infusion. These rapid achievement and high proportion of minimal manifestations and improvement in patients with a refractory course were not reported in previous studies of immunosuppressants and biological agents.^8^^,^^18^, ^19^, ^20^ Notably, we observed negative conversion of acetylcholine receptor antibodies in eight patients. These findings suggested that dual-targeted CAR T-cell therapy may achieve deep and sustained clinical responses through comprehensive B-cell lineage exhaustion, offering the possibility of resetting the pathological immune status.

The safety of our anti-BCMA/CD19 CAR T cells revealed both similarities and distinctions compared to other trials. Like the anti-BCMA/CD19 CAR T cells in systemic lupus erythematosus, we observed a lower incidence of grade 3 or worse hematologic toxicities (except for lymphopenia mainly due to chemotherapy) comparing our previous CAR T-cell trials in multiple myeloma.^16^^,^^21^ The incidence of cytokine release syndrome was 39%, which was lower than those in systemic lupus erythematosus (90%) and in multiple myeloma (92%).^16^^,^^17^ Another study summarized 18 patients with autoimmune diseases who received CD19 CAR T-cell therapy, among which 14 patients (78%) experienced low-grade CRS reactions (13 of them were grade 1).^22^ The difference of CRS in autoimmune diseases from that in hematology may be due to the fact that in B-cell malignancies, patients often have a massive tumor burden, providing a vast antigenic load that triggers robust activation and expansion of CAR-T cells after infusion. In contrast, myasthenia gravis is a disease driven by autoreactive B-cells and plasma cells, which, while pathogenic, represent fewer CAR-T target antigen-positive cells. This may explain the more controlled and milder CRS. In addition, no ICANS occurred in our study.

Currently, two other teams have reported two case reports of autologous CAR T-cell therapy for myasthenia gravis.^11^^,^^13^ One targets BCMA and the other CD19. For anti-CD19 CAR T cells, circulating CD19+ B cells cleared on day 8; for anti-BCMA CAR T cells, it was within seven days. Among three participants in these two studies, only one patient had grade 1 CRS, showing good safety, which is in line with our results.^11^^,^^13^ The trial of anti-BCMA RNA CAR T cells (rCAR-T) in myasthenia gravis in part 2 showed that 89% of participants had ≥2-point improvement in MG-ADL score, and 56% had ≥6-point improvement.^12^ With similar baselines, a higher proportion of participants had ≥6-point improvement in MG-ADL in our study. No cytokine release syndrome was observed in the rCAR-T trial, but the effectiveness of rCAR-T seems to be more dependent on repeated infusions.^12^

However, the prolonged B-cell aplasia and high rate of hypogammaglobulinemia likely reflect the impact of CD19/BCMA bispecific targeting on pro-B cells to long-lived plasma cells and bring a possible high risk of infection. This expanded immunosuppressive effect must be weighed against the clinical benefits, particularly when compared to the more limited B-cell depletion achieved by rituximab and inebilizumab or the transient IgG reduction from neonatal Fc receptor inhibitors.^23^ Even so, the incidence of lower respiratory tract infections is not very high (17%), suggesting that the hypogammaglobulinemia may be clinically manageable with appropriate monitoring and replacement therapy by low-dose intravenous immunoglobulin when indicated.

The temporal patterns observed in our study provide a novel insight into the relationship between B-cell depletion and clinical improvement in myasthenia gravis. The rapid depletion of circulating CD19+ B cells occurred in most patients on day 7 preceded the delayed clinical improvement, which occurred after 14 days post-CAR T-cell infusion. Notably, although B-cell reconstitution was observed in ten participants by 180 days after infusion, both clinical improvement and reduction of acetylcholine receptor antibodies maintained, suggesting that CAR-T therapy may facilitate immune “resetting” rather than requiring continuous B-cell aplasia, this situation were also reported by previous studies.^13^^,^^17^^,^^24^

This dose-escalation design identified no dose-limiting toxicity, and the incidence of grade 3-4 adverse events was similar among three dose levels, while the incidence of cytokine release syndrome was higher at dose level 2 (83%). Given that all doses of CAR T cells in our study showed expected efficacy, the optimal balance between efficacy and safety remains hard to define. Perhaps for autoimmune diseases, low doses of CAR-T cells may provide a better risk-benefit ratio. However, this needs further research. Besides, whether the 56% B-cell reconstitution without symptom progression in our study represents a immune reset still needs to be addressed through longer follow-up, as well as analysis of the B-cell profile, function, and bone marrow cytological analysis.

The limitations of our study include the short follow-up period, the single-arm and single-center design, and the small sample size. These limitations restrict the assessment of long-term durability, as well as the statistical power and generalizability of our findings. The exclusion of anti-muscle-specific kinase antibody patients raises questions regarding the generalizability to other myasthenia gravis subtypes. In addition, the absence of male participants in dose level 2, a consequence of sequential enrollment, limits the interpretation of safety from this group regarding potential sex-based differences. Moreover, the participants were only recruited from China, which may restrict the generalisability of the results to diverse race settings. Lastly, systematic assessment of vaccine-induced antibody titers and the development of structured revaccination protocols remain insufficiently established.

In conclusion, anti-BCMA/CD19 bispecific CAR T cells show a good safety and efficacy in patients with refractory myasthenia gravis. Most patients achieved minimal manifestation status and discontinued glucocorticoids and non-steroidal immunosuppressants, with approximately half showing seroconversion of anti-AChR antibodies. Considering both adverse events and activity, a dose of 1.0 × 10^6^ CAR T cells per kg is recommended for future trials. Further studies with larger cohorts and longer follow-up are needed to assess long-term outcomes and late toxicities. This study supports expanding CAR T-cell therapy from oncology to autoimmune diseases and suggests its potential for immune reset in myasthenia gravis.

## Contributors

YZ, MS, JZ, GC, ZZ, XH, JC, SL, and GQ contributed to the study design, data analysis and interpretation, and manuscript writing. ZY, HC, WC, FZ, KQ, DL, WS, HL, HZ, TQ, DY, WG, JQ, and WZ enrolled and treated patients. MS, DL, GW, and HL contributed to the vector design and developed the anti-BCMA/CD19 CAR T cells. MS and DL contributed to the manufacturing and quality control of the CAR T cells. ZZ, SZ, WZ, HC, XD, ZW, MY, TL, XG, TM, and DP contributed to data collection and assembly. SZ contributed to the detection of antibody titers. All authors had full access to all the data in the study, approved the final version of the manuscript, and had final responsibility for the decision to submit it for publication. YZ and MS had accessed and verified the data.

## Data sharing statement

The study protocol and the data supporting the findings are included in the supplementary appendix. The participant data that underpin the results may be shared with qualified researchers upon a reasonable request to the corresponding author.

## Declaration of interests

All authors declare no competing interests.
