# Supplementary material for: Anti-BCMA/CD19 CAR T cell therapy in patients with refractory generalized myasthenia gravis: a single-arm, phase 1 trial
Source: eClinicalMedicine. 2025 Oct 31;90:103621. doi: 10.1016/j.eclinm.2025.103621 (PMC12615342; doi:10.1016/j.eclinm.2025.103621)
Supplement: Supplementary Material 1 [file mmc1.docx]

# Protocol

**Safety and Efficacy of Anti-BCMA/CD19 Bispecific CAR T-Cell Therapy in the Treatment of Autoimmune Diseases**

**PI/Department:** Jiang Cao, M.D. & Ph.D./Department of hematology

**Study sponsor and monitor:** The Affiliated Hospital of Xuzhou Medical University

**Study duration:** 2022.03-2025.03

**Version:** V1.0

**Protocol updated:** 2022.02.10

**1.Abstract**

| **Title** | Safety and Efficacy of Anti-BCMA/CD19 Bispecific CAR T-Cell Therapy in the Treatment of Autoimmune Diseases |
| --- | --- |
| **Phase** | Phase 1 |
| **Objectives** | **Objectives for refractory myasthenia gravis (MG):**  The primary objectives for refractory MG:  To evaluate the safety of anti-BCMA/CD19 bispecific CAR T cells , including DLT, MTD, and the incidence and severity of cytokine release syndrome (CRS), immune effector cell-associated neurotoxicity syndrome (ICANS), damage to major organs (heart, liver, and kidney), and vital signs.  The secondary objectives for refractory MG:  To evaluate the changes in Myasthenia Gravis Activities of Daily Living (MG-ADL) score, Quantitative Myasthenia Gravis (QMG) score, Myasthenia Gravis Quality of Life 15-revised (MG-QoL-15r) score from baseline at each follow-up assessment up to 36 months.  The predefined exploratory objectives for refractory MG:  To evaluate the proportion of participants achieving minimal symptom expression (MSE, defined as a MG-ADL score of 0 or 1), changes in serum anti-AChR antibodies titer, and the proportion of participants whose glucocorticoid dosage was tapered to 5 mg or less per day.  **Objectives for relapsed/refractory neuromyelitis optica spectrum disorder (NMOSD):**  The primary objectives for relapsed/refractory NMOSD:  To evaluate the safety of anti-BCMA/CD19 bispecific CAR T cells , including DLT, MTD, and the incidence and severity of CRS, ICANS, damage to major organs, and vital signs.  The secondary objectives for relapsed/refractory NMOSD:  To evaluate the changes of pathogenic antibody titers (aquaporin-4 antibodies) in peripheral blood or cerebrospinal fluid and expanded disability status scale (EDSS) score from baseline at each follow-up assessment up to 36 months.  The predefined exploratory objectives for relapsed/refractory NMOSD:  The annualized relapse rate (ARR), changes in the number of accumulated total active magnetic resonance imaging (MRI) lesions, and visual acuity after CAR T-cell infusion.  **Objectives for refractory chronic inflammatory demyelinating polyneuropathy (CIDP):**  The primary objectives for refractory CIDP:  To evaluate the safety of anti-BCMA/CD19 bispecific CAR T cells , including DLT, MTD, and the incidence and severity of CRS, ICANS, damage to major organs, and vital signs.  The secondary objectives for refractory CIDP:  To evaluate the changes of Inflammatory Neuropathy Cause and Treatment (INCAT) score, INCAT-sensory score, Medical Research Council (MRC) muscle function score, 10m Walk Test (10MWT) performance, and nerve conduction test results from baseline at each follow-up assessment up to 36 months.  **Objectives for systemic lupus erythematosus (SLE):**  The primary objectives for refractory SLE:  To evaluate the safety of anti-BCMA/CD19 bispecific CAR T cells , including DLT, MTD, and the incidence and severity of CRS, ICANS, damage to major organs, and vital signs.  The secondary objectives for SLE:  To evaluate the changes of SLE Disease Activity Index 2000 (SLEDAI-2K) score and the proportion of participants who achieve low disease activity status (LLDAS) from baseline at each follow-up assessment up to 36 months.  The predefined exploratory objectives for SLE:  To evaluate the changes in the levels of SLE-associated serum autoantibodies from baseline at each follow-up assessment up to 36 months. |
| **Study design** | This is a single-center, open-label, and single-arm study to assess the safety and efficacy of anti-BCMA/CD19 bispecific CAR T cells for autoimmune diseases.  Study intervention:  In the dose escalation phase, the following dose of anti-BCMA/CD19 bispecific CAR T cells are planned:   1. Level 1: 1.0 × 10^6^ CAR+ cells/kg 2. Level 2: 3.0 × 10^6^ CAR+ cells/kg 3. Level 3: 5.0 × 10^6^ CAR+ cells/kg   In the dose expansion phase, the recommended dose obtained from the dose escalation phase will be administered. |
| **Number of participants** | Approximately 18 participants will be enrolled into this study |
| **Summary of eligibility criteria** | **Inclusion criteria:**   1. Male or female participants aged ≥ 5 and ≤ 70 years at time of screening. 2. Life expectancy >12 weeks. 3. Diagnosed as an autoimmune disease through physical examination, pathological examination, laboratory tests and imaging, and with poor response to conventional treatment and no effective treatment options. 4. Eastern Cooperative Oncology Group (ECOG) performance status of 0 or 1. 5. Alanine aminotransferase/aspartate aminotransferase < 3 times the upper limit of normal. 6. Total bilirubin < 2.0 mg/dl. 7. Serum creatinine < 2.5 mg/dl. 8. Participants have appropriate peripheral venous access and no contraindications for apheresis. 9. Informed Consent/Assent: All subjects could understand and the willingness to sign a written informed consent.   **Exclusion criteria:**   1. Female who are pregnant or breastfeeding, or female who have plans to become pregnant within one year. 2. Active infections include human immunodeficiency virus, hepatitis B virus, hepatitis C virus, and mycobacterium tuberculosis. 3. Participated in other clinical trials and did not exceed the elution period. 4. Abnormal vital signs and failure to cooperate with the examination. 5. Highly allergic constitution or history of severe allergies, especially to interleukin-2. 6. Any mental or psychological illnesses that could potentially interfere with the completion of treatment. 7. Drug abuse/addiction. 8. Severe systemic or local infections require anti-infective treatment. 9. Evidence of current uncontrolled dysfunction of heart, lung, brain, kidney and other important organs. 10. Other conditions that are not eligible for the trial are in the judgment of the principal investigator.   **Extra criteria for refractory MG:**  Inclusion criteria:   1. Participants must have a confirmed diagnosis of myasthenia gravis and have generalized myasthenia gravis. 2. Participants must have refractory myasthenia gravis. 3. Participants have been positive for serum acetylcholine receptor (AChR) antibodies tested by cell-based assay or radioimmunoassay. 4. Myasthenia Gravis Foundation of America classification of II, III, or IV. 5. Myasthenia Gravis Activities of Daily Living Scale (MG-ADL) score ≥ 6, with more than 50% of the score attributed to non-ocular items. 6. Quantitative Myasthenia Gravis scale (QMG) score ≥ 8, with more than 50% of the score attributed to non-ocular items. 7. The maximum permitted dose was equivalent to 40 mg per day of prednisone. The dose must be stable for a minimum of 4 weeks before lymphodepleting chemotherapy (day -5). 8. The dose of non-steroidal immunosuppressants (including azathioprine, tacrolimus, mycophenolate mofetil, and cyclophosphamide) must be stable for a minimum of 3 months before lymphodepleting chemotherapy (day -5). 9. The dose of pyridostigmine must be stable for a minimum of 1 week before lymphodepleting chemotherapy (day -5).   Exclusion criteria:   1. Using intravenous immunoglobulin, plasma exchange, neonatal Fc receptor inhibitors, or complement inhibitors within 4 weeks before lymphodepleting chemotherapy (day -5). 2. Thymectomy or using of B-cell clearance therapy within 3 months before lymphodepleting chemotherapy (day -5).   **Extra criteria for relapsed/refractory NMOSD:**  Inclusion criteria:   1. Aged 18 to 75 years inclusive, regardless of gender. 2. Participants must meet the 2015 International Panel for NMO Diagnosis (IPND) diagnostic criteria for NMOSD, with confirmed AQP4-IgG seropositivity as per the standard definition, and fulfill the following conditions: (1) inadequate response to at least one immunosuppressant administered for over one year; (2) documented clinical evidence of at least 2 relapses within the past 12 months or 3 relapses within the past 24 months, including at least one relapse in the 12 months prior to screening.   Exclusion criteria:  Treatment with alemtuzumab within 6 months prior to apheresis, or fludarabine or cladribine within 3 months prior to apheresis.  **Extra criteria for refractory CIDP:**  Inclusion criteria:   1. Aged 18 to 75 years inclusive, regardless of gender; 2. A confirmed diagnosis of CIDP according to the 2021 European Academy of Neurology/Peripheral Nerve Society (EAN/PNS) diagnostic criteria; 3. An INCAT disability scale score between 2 and 9; 4. Either of the following treatment-related conditions:  1) Poorly controlled symptoms despite receiving at least one first-line therapy (corticosteroids, intravenous immunoglobulin, or plasma exchange) administered in a standardized manner for a minimum of 3 months;  2) Inability to tolerate corticosteroids, intravenous immunoglobulin, or plasmapheresis due to side effects or other medical contraindications.   Exclusion criteria:   1. Diagnosis of pure sensory atypical CIDP. 2. Polyneuropathy due to other causes, including but not limited to: multifocal motor neuropathy; monoclonal gammopathy of uncertain significance associated with anti-myelin-associated glycoprotein immunoglobulin M antibodies; hereditary demyelinating neuropathy; polyneuropathy attributed to diabetes mellitus; drug- or toxin-induced polyneuropathy, etc. 3. History of myelopathy or evidence of central demyelination. 4. Current or previous (within 12 months prior to screening) alcohol, substance, or prescription medication abuse. 5. Receipt of specific treatments within defined periods prior to lymphodepleting chemotherapy: 6. Within 3 months: plasma exchange, immunoadsorption, Fc-containing therapeutics, or any investigational product; 7. Within 3 months: rituximab, any monoclonal antibody. 8. Any other known autoimmune disease that, in the investigator’s judgment, may confound the evaluation of CIDP-related clinical symptoms.   **Extra criteria for SLE:**  Inclusion criteria:   1. Aged 18 to 75 years inclusive, regardless of gender, with a body weight of no less than 35 kg. 2. Diagnosis of active SLE, meeting at least 4 of the 11 criteria revised by the American College of Rheumatology (ACR) in 1997. 3. Participants must be evaluated using the SLE Disease Activity Index 2000 (SLEDAI-2K), with a total score ≥10, indicating moderate to severe disease activity.   Exclusion criteria:   1. Severe lupus nephritis within the last 2 months (defined as urinary protein >6 g/24 hours, or serum creatinine >2.5 mg/dL [221 μmol/L]), or requiring hemodialysis, or receipt of high-dose corticosteroids (prednisone >100 mg/day or equivalent) for ≥14 days. 2. Central nervous system (CNS) involvement (including epilepsy, psychosis, organic brain syndrome, cerebrovascular accident, encephalitis, or CNS vasculitis) attributable or not attributable to SLE within the last 2 months. 3. Participation in any other clinical trial within 28 days prior to initial screening or within 5 times the half-life of the investigational compound (whichever is longer). 4. Use of tumor necrosis factor (TNF) inhibitors or interleukin receptor blockers within the past year. 5. Use of intravenous immunoglobulin (IVIG), plasma exchange, or prednisone ≥100 mg/day for ≥14 days within the past month. |
| **Statistical methods** | Statistical Package for Social Sciences 26.0 software for Windows (SPSS, Chicago, IL, USA) for statistical analysis. Categorical variables were expressed as numbers (percentages), normally distributed continuous variables were expressed as mean and standard deviation (SD), non-normally distributed variables continuous were expressed as median and interquartile range (IQR). |
| **Study duration** | The duration required to complete this study will be contingent upon the number of patients. We anticipate that it will take approximately four years to conclude this trial. This study will be partitioned into two phases. The first phase involves screening and enrolling patients for CAR T-cell infusion to assess efficacy and safety, while the second phase entails a three-year long-term follow-up. |

**2.****Background**

Autoimmune diseases constitute a heterogeneous group of disorders characterized by diverse clinical manifestations. Although the phenotype expression of these diseases varies widely, their underlying pathogenesis universally involves immune responses directed against self-antigens. Autoimmune reactions, either as primary triggers or contributing factors, are implicated in more than 80 distinct human diseases. Immunopathological damage may be restricted to a single organ, as seen in autoimmune thyroid diseases, or may involve multiple organ systems, as in the case of systemic lupus erythematosus (SLE). These diseases affect individuals across all genders, age groups, ethnicities, races, and socioeconomic strata, albeit with varying prevalence rates. Current therapeutic strategies for autoimmune diseases primarily aim to correct specific immune deficiencies or modulate the overall immune response. In cases where the autoimmune injury is localized and less severe, functional replacement therapy may be effective-for example, administration of thyroxine in Hashimoto's thyroiditis can alleviate associated symptoms. However, in most cases, the immunopathological damage is extensive, necessitating systemic interventions. Despite advances in immunology, existing treatment modalities remain suboptimal, highlighting the urgent need for more effective and targeted therapeutic approaches.

It is well-known that B cells originate from hematopoietic stem cells. Lymphoid stem cells differentiate and develop into mature B cells through a series of stages within the bone marrow. Subsequently, these mature B cells enter the peripheral lymphoid tissue and ultimately differentiate into plasma cells capable of secreting immunoglobulins. In the development of autoimmune diseases, B cells not only produce autoantibodies but also function as antigen-presenting cells and regulate other immune cells. Consequently, the depletion of B cells has gradually emerged as a potential therapeutic strategy. B cells possess numerous surface molecules on their membranes, including CD19 and CD20. These molecules are present on primary B cells and pre-B cells and remain on the surface of B cells throughout all stages until mature B cells are externally transformed into plasma cells. Rituximab, a murine-human chimeric monoclonal antibody targeting the B-cell specific antigen CD20, can selectively and significantly deplete B cells. It has been extensively used in the treatment of B-cell lymphoma, yielding satisfactory therapeutic outcomes. Currently, many scholars have experimentally applied this therapy to treat refractory SLE and other autoimmune diseases such as rheumatoid arthritis (RA), and the therapeutic effects are promising.^3-5^

Chimeric antigen receptor T-cell (CAR T) therapy represents one of the most significant recent advancements in bio-medicine. This therapy modifies immune T cells in vitro using biotechnology, enabling them to recognize antigens on the surface of cancer cells and achieving the targeted killing of cancer cells. First reported by Zelig Eshhar in 1989,^6^ CAR T therapy faced various limitations in its early days and thus remained in a dormant state. Since 2010, however, this technology has witnessed rapid development. A series of clinical trials have confirmed that CAR T-cell therapy can effectively treat various refractory and recurrent B-cell malignant hematological disorders, including B-cell acute lymphoblastic leukemia (B-ALL), chronic lymphocytic leukemia, lymphoma, etc.^7-10^ Based on the current clinical trial results, the efficacy of CD19 CAR T cells in treating relapsed or refractory B-ALL is the most conclusive. In February 2018, Maude et al. published the results of the phase 2 clinical trial of the marketed CAR-T drug “Tisagenlecleucel”.^11^ Seventy-five patients aged 3 to 21 with relapsed or refractory B-ALL received treatment. The complete remission rate within 3 months of treatment was 81%. The 6-month event-free survival rate and overall survival rate were 73% and 90% respectively, while the 12-month event-free survival rate and overall survival rate were 50% and 76% respectively. This novel therapy offers great hope of survival to patients with relapsed or refractory B-cell malignant hematological tumors.

B-cell maturation antigen (BCMA), also referred to as CD269, belongs to the tumor necrosis factor receptor family. It is capable of binding to two ligands, namely the B cell activating factor (BAFF) or the a proliferation-inducing ligand (APRIL). BCMA is expressed on the surface of normal mature B cells and plasma cells. Moreover, it is highly expressed on the surface of malignant plasma cells. Precisely due to the expression characteristics of BCMA, numerous research centers are currently conducting clinical trials of BCMA CAR-T for the treatment of multiple myeloma (MM). The bb2121, developed in the United States and undergoing Phase I, II, and III clinical studies globally, is a murine anti-BCMA CAR T. The results of Phase I were reported. A total of 33 patients with relapsed/refractory (R/R) MM were enrolled. The overall response rate was 85%, the strict complete response (sCR) and complete remission (CR) rate was 45%, and the median progression-free survival (PFS) was 11.8 months [10]. A total of 128 patients with R/R MM were recruited for the Phase II clinical study. The overall response rate was 73%, and the median PFS was 8.8 months.^13^ Given the superior therapeutic effect of this drug, idecabtagene vicleucel (bb2121) was approved for marketing in the United States in March 2021, becoming the first CAR T-cell novel drug targeting BCMA.

The first CAR T cells for MM developed in China, LCAR-B38M, has two heavy chain variable regions targeting BCMA from alpaca in its CAR structure. In a Phase I clinical study, 57 patients with R/R MM were recruited at the Second Affiliated Hospital of Xi'an Jiaotong University. The overall response rate was 88%, the sCR and CR rates were 74%, and the median PFS was 15 months [12]. In a multicenter study reported by Ruijin Hospital in Shanghai, 17 patients with R/R MM were enrolled. The overall response rate was 88%, the rate of responses above CR was 76%, and the 12-month PFS rate was 52.9%.^15^ A multi-center Phase I/II study in the United States enrolled 97 patients with R/R MM. The overall response rate was 97%, the rate of responses above CR was 67%, and the 12-month PFS rate was 77%.^16^ LCAR-B38M is anticipated to be the first CAR-T product for MM to be launched in China.

Based on the pathogenesis of autoimmune diseases and the remarkable effects of CD19 and BCMA CAR T cells in eliminating B cells and plasma cells, this study aims to observe the safety and efficacy of sequential infusion of anti-BCMA/CD19 bispecific CAR T cells in patients with refractory autoimmune diseases. The clinical data obtained from this study can offer data support for novel treatment regimens of refractory autoimmune diseases.

1. **Objectives**

3.1 Objectives for refractory myasthenia gravis (MG):

3.1.1 The primary objectives for refractory MG:

To evaluate the safety of anti-BCMA/CD19 bispecific CAR T cells , including DLT, MTD, and the incidence and severity of cytokine release syndrome (CRS), immune effector cell-associated neurotoxicity syndrome (ICANS), damage to major organs (heart, liver, and kidney), and vital signs.

3.1.2 The secondary objectives for refractory MG:

To evaluate the changes in Myasthenia Gravis Activities of Daily Living (MG-ADL) score, Quantitative Myasthenia Gravis (QMG) score, Myasthenia Gravis Quality of Life 15-revised (MG-QoL-15r) score from baseline at each follow-up assessment up to 36 months.

3.1.3 The predefined exploratory objectives for refractory MG:

To evaluate the proportion of participants achieving minimal symptom expression (MSE, defined as a MG-ADL score of 0 or 1), changes in serum anti-AChR antibodies titer, and the proportion of participants whose glucocorticoid dosage was tapered to 5 mg or less per day.

3.2 Objectives for relapsed/refractory neuromyelitis optica spectrum disorder (NMOSD):

3.2.1 The primary objectives for relapsed/refractory NMOSD:

To evaluate the safety of anti-BCMA/CD19 bispecific CAR T cells , including DLT, MTD, and the incidence and severity of CRS, ICANS, damage to major organs, and vital signs.

3.2.2 The secondary objectives for relapsed/refractory NMOSD:

To evaluate the changes of pathogenic antibody titers (aquaporin-4 antibodies) in peripheral blood or cerebrospinal fluid and expanded disability status scale (EDSS) score from baseline at each follow-up assessment up to 36 months.

3.2.3 The predefined exploratory objectives for relapsed/refractory NMOSD:

The annualized relapse rate (ARR), changes in the number of accumulated total active magnetic resonance imaging (MRI) lesions, and visual acuity after CAR T-cell infusion.

3.3 Objectives for refractory chronic inflammatory demyelinating polyneuropathy (CIDP):

3.3.1 The primary objectives for refractory CIDP:

To evaluate the safety of anti-BCMA/CD19 bispecific CAR T cells , including DLT, MTD, and the incidence and severity of CRS, ICANS, damage to major organs, and vital signs.

3.3.2 The secondary objectives for refractory CIDP:

To evaluate the changes of Inflammatory Neuropathy Cause and Treatment (INCAT) score, INCAT-sensory score, Medical Research Council (MRC) muscle function score, 10m Walk Test (10MWT) performance, and nerve conduction test results from baseline at each follow-up assessment up to 36 months.

3.4 Objectives for systemic lupus erythematosus (SLE):

3.4.1 The primary objectives for refractory SLE:

To evaluate the safety of anti-BCMA/CD19 bispecific CAR T cells , including DLT, MTD, and the incidence and severity of CRS, ICANS, damage to major organs, and vital signs.

3.4.2 The secondary objectives for SLE:

To evaluate the changes of SLE Disease Activity Index 2000 (SLEDAI-2K) score and the proportion of participants who achieve low disease activity status (LLDAS) from baseline at each follow-up assessment up to 36 months.

3.4.3 The predefined exploratory objectives for SLE:

To evaluate the changes in the levels of SLE-associated serum autoantibodies from baseline at each follow-up assessment up to 36 months.

1. **Study design**
   1. **Study design**

This study adopted an open-label, single-arm study to evaluate the safety and efficacy of anti-BCMA/CD19 bispecific CAR T cells in the treatment of autoimmune diseases.

The study is conducted using the dose escalation method and the observation period of DLT is within 28 days after CAR-T cell infusion. The detailed dose escalation plan is as follows:

(1) In the dose escalation stage, a total of three dose groups were designed: 1) Dose level 1: 1.0×10^6^ CAR+ cells/kg; 2) Dose level 2: 3.0×10^6^ CAR+ cells/kg; 3) Dose level 3: 5.0×10^6^ CAR+ cells/kg. Each dosage group is planned to include six participants.

(2) Starting from a low dose, after the observation period of each dose group ended, the researchers evaluated the toxic reactions and DLT to determine whether to increase to the next high dose or stop the study.

(3) If none of the six subjects in the same dose group developed DLT during the observation period, they would be incremented to the next high-dose group study.

(4) Among the six subjects in the same dose group, 1 case developed DLT during the observation period. Then, six subjects in this dose group were added for the study at the same dose. If no DLT occurred among the added six subjects, the study was incremented to the next high-dose group. If one or more of the three additional subjects develop DLT, it will be reduced to the previous low-dose group.

(5) Among the six subjects in the same dose group, if more than 1 case developed DLT during the observation period, it would be reduced to the previous low-dose group.

(6) When decreasing to the previous low-dose group, if there are only six subjects in this dose group, add another six subjects for the trial. If there are already 6 subjects with this dose, then this dose is the MTD.

(7) If no DLT occurs after the dose is increased to the set maximum dose of 5.0×10^6^ CAR+ cells/kg, the researchers will discuss the existing safety data, including parameters such as possible DLT, to determine whether to perform dose increase, etc.

- 1. **Definition of dose-limiting toxicity**

Any CAR T cells related grade ≥ 3 toxicity occurred within 28 days of infusion, except for the following situations:

(1) Grade 3 cytokine release syndrome (CRS) that resolved with appropriate medical intervention within 3 days (recovery to grade ≤ 2).

(2) Grade 3 to 4 tumor lysis syndrome (TLS) lasting ≤ 7 days.

(3) Hematological toxicity: 1) Grade 3 neutropenia at any time or grade 4 neutropenia lasting ≤ 14 days; 2) Grade 3 anemia at any time or grade 4 anemia lasting ≤ 14 days; 3) Grade 3 thrombocytopenia at any time or grade 4 thrombocytopenia lasting for ≤ 21 days; 4) All cytopenia except for the neutropenia, anemia, and thrombocytopenia.

(4) Non-hematological toxicity: 1) Fever of any grade, including febrile neutropenia; 2) Grade 3 diarrhea lasting ≤ 72 hours; 3) Grade 3 nausea and/or vomiting lasting ≤ 72 hours; 4) Grade 3 fatigue lasting ≤ 7 days; 5) Grade 3-4 elevations in transaminases, bilirubin, creatinine kinase, blood urea nitrogen (BUN), or creatinine persisting for ≤ 7 days; 6) Asymptomatic lipase elevation without clinical symptoms or signs of pancreatitis; 7) Any asymptomatic, non-hematological, grade 3 acute laboratory abnormalities that are rapidly reversible (returning to baseline or ≤ grade 2 within 7 days).

- 1. **Definition of Maximum tolerated dose (MTD)**

MTD is defined as the highest dose at which dose-limiting toxicity (DLT) occurs in no more than 1 out of 6 subjects within 28 days following cell infusion.

- 1. **Definition of the end of study**

The end of the study is defined as the point at which the last visit of the final subject has been completed, the subject is lost to follow-up, or the subject withdraws from the study (including in the case of death).

- 1. **Sample**

Approximately 18 participants will be enrolled in this study.

- 1. **Study duration**

The duration required to complete this study will be contingent upon the number of patients. We anticipate that it will take approximately four years to conclude this trial. This study will be partitioned into two phases. The first phase involves screening and enrolling patients for CAR T-cell infusion to assess efficacy and safety, while the second phase entails a three-year long-term follow-up. The study protocol permitted an interim analysis upon reaching the primary endpoints of establishing the maximum tolerated dose with nodose-limiting toxicities and assessing preliminary efficacy.

1. **Study population**
   1. **Enrollment of participants**

5.1.1 Inclusion criteria:

(1) Male or female participants aged ≥ 5 and ≤ 70 years at time of screening.

(2) Life expectancy > 12 weeks.

(3) Diagnosed as an autoimmune disease through physical examination, pathological examination, laboratory tests and imaging, and with poor response to conventional treatment and no effective treatment options.

(4) Eastern Cooperative Oncology Group (ECOG) performance status of 0 or 1.

(5) Alanine aminotransferase/aspartate aminotransferase < 3 times the upper limit of normal.

(6) Total bilirubin < 2.0 mg/dl.

(7) Serum creatinine < 2.5 mg/dl.

(8) Participants have appropriate peripheral venous access and no contraindications for apheresis.

(9) Informed Consent/Assent: All subjects could understand and the willingness to sign a written informed consent.

5.1.2 Exclusion criteria:

(1) Female who are pregnant or breastfeeding, or female who have plans to become pregnant within one year.

(2) Active infections include human immunodeficiency virus, hepatitis B virus, hepatitis C virus, and mycobacterium tuberculosis.

(3) Participated in other clinical trials and did not exceed the elution period.

(4) Abnormal vital signs and failure to cooperate with the examination.

(5) Highly allergic constitution or history of severe allergies, especially to interleukin-2.

(6) Any mental or psychological illnesses that could potentially interfere with the completion of treatment.

(7) Drug abuse/addiction.

(8) Severe systemic or local infections require anti-infective treatment.

(9) Evidence of current uncontrolled dysfunction of heart, lung, brain, kidney and other important organs.

(10) Other conditions that are not eligible for the trial are in the judgment of the principal investigator.

5.1.3 Extra criteria for refractory MG:

5.1.3.1 Extra inclusion criteria:

(1) Participants must have a confirmed diagnosis of myasthenia gravis and have generalized myasthenia gravis.

(2) Participants must have refractory myasthenia gravis.

(3) Participants have been positive for serum acetylcholine receptor (AChR) antibodies tested by cell-based assay or radioimmunoassay.

(4) Myasthenia Gravis Foundation of America classification of II, III, or IV.

(5) Myasthenia Gravis Activities of Daily Living Scale (MG-ADL) score ≥ 6, with more than 50% of the score attributed to non-ocular items.

(6) Quantitative Myasthenia Gravis scale (QMG) score ≥ 8, with more than 50% of the score attributed to non-ocular items.

(7) The maximum permitted dose was equivalent to 40 mg per day of prednisone. The dose must be stable for a minimum of 4 weeks before lymphodepleting chemotherapy (day -5).

(8) The dose of non-steroidal immunosuppressants (including azathioprine, tacrolimus, mycophenolate mofetil, and cyclophosphamide) must be stable for a minimum of 3 months before lymphodepleting chemotherapy (day -5).

(9) The dose of pyridostigmine must be stable for a minimum of 1 week before lymphodepleting chemotherapy (day -5).

5.1.3.2 Extra exclusion criteria:

(1) Using intravenous immunoglobulin, plasma exchange, neonatal Fc receptor inhibitors, or complement inhibitors within 4 weeks before lymphodepleting chemotherapy (day -5).

(2) Thymectomy or using of B-cell clearance therapy within 3 months before lymphodepleting chemotherapy (day -5).

5.1.4 Extra criteria for relapsed/refractory NMOSD:

5.1.4.1 Extra inclusion criteria:

(1) Aged 18 to 75 years inclusive, regardless of gender.

(2) Participants must meet the 2015 International Panel for NMO Diagnosis (IPND) diagnostic criteria for NMOSD, with confirmed AQP4-IgG seropositivity as per the standard definition, and fulfill the following conditions: 1) inadequate response to at least one immunosuppressant administered for over one year; 2) documented clinical evidence of at least 2 relapses within the past 12 months or 3 relapses within the past 24 months, including at least one relapse in the 12 months prior to screening.

5.1.4.2 Extra exclusion criteria:

Treatment with alemtuzumab within 6 months prior to apheresis, or fludarabine or cladribine within 3 months prior to apheresis.

5.1.5 Extra criteria for refractory CIDP:

5.1.5.1 Extra inclusion criteria:

(1) Aged 18 to 75 years inclusive, regardless of gender;

(2) A confirmed diagnosis of CIDP according to the 2021 European Academy of Neurology/Peripheral Nerve Society (EAN/PNS) diagnostic criteria;

(3) An INCAT disability scale score between 2 and 9;

(4) Either of the following treatment-related conditions:

1) Poorly controlled symptoms despite receiving at least one first-line therapy (corticosteroids, intravenous immunoglobulin, or plasma exchange) administered in a standardized manner for a minimum of 3 months;

2) Inability to tolerate corticosteroids, intravenous immunoglobulin, or plasmapheresis due to side effects or other medical contraindications.

5.1.5.2 Exclusion criteria:

(1) Diagnosis of pure sensory atypical CIDP.

(2) Polyneuropathy due to other causes, including but not limited to: multifocal motor neuropathy; monoclonal gammopathy of uncertain significance associated with anti-myelin-associated glycoprotein immunoglobulin M antibodies; hereditary demyelinating neuropathy; polyneuropathy attributed to diabetes mellitus; drug- or toxin-induced polyneuropathy, etc.

(3) History of myelopathy or evidence of central demyelination.

(4) Current or previous (within 12 months prior to screening) alcohol, substance, or prescription medication abuse.

(5) Receipt of specific treatments within defined periods prior to lymphodepleting chemotherapy:

1. Within 3 months: plasma exchange, immunoadsorption, Fc-containing therapeutics, or any investigational product;
2. Within 3 months: rituximab, any monoclonal antibody.

(6) Any other known autoimmune disease that, in the investigator’s judgment, may confound the evaluation of CIDP-related clinical symptoms.

5.1.6 Extra criteria for SLE:

5.1.6.1 Extra inclusion criteria:

(1) Aged 18 to 75 years inclusive, regardless of gender, with a body weight of no less than 35 kg.

(2) Diagnosis of active SLE, meeting at least 4 of the 11 criteria revised by the American College of Rheumatology (ACR) in 1997.

(3) Participants must be evaluated using the SLE Disease Activity Index 2000 (SLEDAI-2K), with a total score ≥10, indicating moderate to severe disease activity.

5.1.6.2 Extra exclusion criteria:

(1) Severe lupus nephritis within the last 2 months (defined as urinary protein >6 g/24 hours, or serum creatinine >2.5 mg/dL [221 μmol/L]), or requiring hemodialysis, or receipt of high-dose corticosteroids (prednisone >100 mg/day or equivalent) for ≥14 days.

(2) Central nervous system (CNS) involvement (including epilepsy, psychosis, organic brain syndrome, cerebrovascular accident, encephalitis, or CNS vasculitis) attributable or not attributable to SLE within the last 2 months.

(3) Participation in any other clinical trial within 28 days prior to initial screening or within 5 times the half-life of the investigational compound (whichever is longer).

(4) Use of tumor necrosis factor (TNF) inhibitors or interleukin receptor blockers within the past year.

(5) Use of intravenous immunoglobulin (IVIG), plasma exchange, or prednisone ≥100 mg/day for ≥14 days within the past month.

- 1. **Lifestyle Precautions**

It is recommended not to smoke, drink alcohol or consume foods or beverages that affect metabolism, such as alcohol, caffeine or tea, at least 48 hours before taking all medications until the last blood draw.

- 1. **Screening failed**

Screening failure was defined as subjects withdrawing from the study before infusion of anti-BCMA/CD19 bispecific CAR T cells after signing the informed consent form. Subjects whose screening failed should fill in the original documents and no further follow-up is required. If a subject fails the screening for special reasons and the researcher deems it possible to re-participate in the research screening, a new subject number will be assigned, and the informed consent form will be re-signed.

- 1. **Participants supplementation criteria:**

During the study period, the subjects withdrew from the study for any reason other than the safety of infusion of anti-BCMA/CD19 bispecific CAR T cells. It was up to the researchers to decide whether to recruit the subjects for supplementation.

1. **Study methodology**
   1. **Dosage and Administration of CAR T cells**

Dose escalation stage: It is recommended that a single intravenous infusion of anti-BCMA/CD19 bispecific CAR T cells be completed within 30 minutes to 1 hour. A total of three dose groups were designed as follows: low-dose Group: the reinfusion dose of anti-BCMA-CD19 CAR-T cells was 1.0×10⁶ CAR+ cells/kg; medium-dose Group: The reinfusion dose of anti-BCMA-CD19 CAR-T cells was 3.0×10⁶ CAR+ cells/kg; high-dose Group: The reinfusion dose of anti-BCMA-CD19 CAR-T cells was 5.0×10⁶ CAR+ cells/kg. Each dose group was planned to include 3 to 6 subjects.

Dose expansion stage: It is recommended that a single intravenous infusion of anti-BCMA/CD19 bispecific CAR T cells be completed within 30 minutes to 1 hour. It is proposed to include at least 6 subjects. The dose in the expansion stage will adopt the recommended dose obtained in the dose escalation stage.

On the fifth day before the infusion of CAR T cells, the participants should receive lymphocyte depletion chemotherapy. The recommended regimen is as follows: cyclophosphamide at 250mg/m²/d (from D-5 to D-3), and fludarabine at 30mg/m²/D (from D-5 to D-3). The specific dosage shall be determined by the researcher according to the actual clinical situation. Before the infusion of anti-BCMA-CD19 CAR-T cells, the researchers may choose to pretreat the cells with acetaminophen and diphenhydramine or other H1-antihistamines to prevent hypersensitivity reactions.

- 1. **Dose Adjustment and Discontinuation Criteria**

Not applicable to this study.

- 1. **Combined Treatment**

All combined medications (including the start/end time and the purpose of medication) must be recorded in the corresponding sections of the subjects' original documents.

- 1. **Study process**

This study encompasses five steps: (1) Recruitment and screening of participants (D-48~D-28); (2) Preparation for CAR T cells, which includes leukocyte apheresis and CAR T cells preparation (D-28~D-6); (3) Lymphodepleting Chemotherapy (D-5~D-3); (4) CAR T cells infusion (D0); (5) Assessment and follow-up.

- - 1. **Recruitment and Screening of Participants**

In the informed consent process, researchers are obliged to elaborate in detail to each participant the purpose, relevant procedures, anticipated duration, potential risks and benefits, as well as any possible discomfort associated with the study. It is essential that each participant is fully aware that their participation in this study is entirely voluntary. Participants reserve the right to withdraw from the study and revoke their informed consent at any time without any adverse impact on their rights and interests. After explaining the fundamental aspects of the study and verifying that each intended participant comprehends the study's objectives, each participant or their legal guardian should be asked to affix their signature, the date, and provide their contact information on the informed consent form. Before signing and dating the form, the participant or their guardian should carefully read and consider the content. Only after thoroughly understanding the research process and making a voluntary decision to participate in the study should they sign the informed consent form. Meanwhile, the researcher should also sign the form. The informed consent form is prepared in duplicate. Individuals who have not given informed consent or have not signed the informed consent form are strictly prohibited from enrolling in the study.

Then, blood samples will be collected and tested for HIV. These samples will then be sent to the CAR T cells production department for an assessment of T cell production feasibility. Approximately 1-2 weeks later, the production department will evaluate the proliferation and transduction efficiency of the participant's T cells in vitro to determine whether their T cells are suitable for large-scale CAR T cells production.

The patients who pass the previous stage will be examined for diseases and a series of routine examinations. The results of the examination items will be collected. According to the Inclusion criteria and exclusion criteria, the suitability of CAR-T cell therapy will be confirmed. Screen tests (D-48~D-28):

(1) Demographic data: Date of birth, gender, ethnicity, age, nationality, etc.

(2) Medical history: Current medical history, past medical history, medication history, allergy history, alcohol consumption history, substance abuse history, blood transfusion history, clinical trial participation history, and previous surgical history, etc.

(3) General condition: height, weight, vital signs (including respiratory rate, pulse, blood pressure, oxyhemoglobin saturation, temperature), and general physical examination.

(4) Blood routine examination: red blood cell count (RBC), hemoglobin (HGB), platelet count (PLT), white blood cell count (WBC), absolute neutrophil count (ANC), and absolute lymphocyte count (ALC).

(5) Blood biochemical examination: albumin (ALB), alanine aminotransferase (ALT), aspartate aminotransferase (AST), alkaline phosphatase (ALP), gamma-glutamyl transferase (GGT), total bilirubin (TBIL), direct bilirubin (DBIL), urea (UREA) or blood urea nitrogen (BUN), creatinine (CREA), creatine kinase (CK), lactate dehydrogenase (LDH), triglycerides (TG), cholesterol (CHOL), high-density lipoprotein cholesterol (HDL-C), low-density lipoprotein cholesterol (LDL-C), blood glucose (GLU), electrolytes (potassium, sodium, chloride, calcium, magnesium, phosphorus), procalcitonin (PCT), and erythrocyte sedimentation rate (ESR).

(6) Coagulation function examination: prothrombin time (PT), international normalized ratio (INR), activated partial thromboplastin time (APTT), thrombin time (TT), and fibrinogen (FIB).

(7) Infectious disease examination: HIV, hepatitis B (HepBS Ab, Hep BS Ag, Hep Be Ab, Hep Be Ag and Hep Bc Ab), and hepatitis C (Hep C Ab).

(8) Lymphocyte subsets: CD3+ T cells, CD4+ T cells, CD8+ T cells, CD19+ B cells, CD16+CD56+ natural killer (NK) cells.

(9)Others: urine test, creatine kinase isoenzyme, troponin, β-human chorionic gonadotropin, ultrasonic cardiogram (UCG), electrocardiograph (ECG), high-sensitivity C-reactive protein (hs-CRP), interleukin 6 (IL-6), immunoglobulin levels (IgG, IgM, and IgA).

- - 1. **CAR T cells production**

Enriched monocytes from patients were further isolated via density gradient centrifugation (Lymphoprep, Axis Shield, Norway) at 800 g for 20 minutes without interruption at room temperature. The interface, the peripheral blood mononuclear cell (PBMC) layer, was collected and washed with 0.9% saline. CD3+ T cells purification and activation were performed using magnetic Dynabeads (Thermo Fisher Scientific) according to the manufacturer’s instructions. At 24 hours after activation, T cells were transduced with CD19 lentivirus (MOI = 5). Briefly, 3×10^6^ T cells were mixed with 100 μL of lentivirus (108 TU/mL) and centrifuged at 800 g for 60 minutes at 30°C. After centrifugation, the cell culture flask was returned to a 37°C and 5% CO_2_ incubator. The transduced T cells were continuously cultured for 5-10 days before the subsequent assay (the production procedure of anti-BCMA/CD19 bispecific CAR T cells refer to previously published study).^17^

- - 1. **Lymphodepleting Chemotherapy**

The aim of lymphodepleting chemotherapy is to deplete the endogenous lymphocytes to facilitate the proliferation of reinfused CAR-T cells. All patients will be pretreated with fludarabine at 30mg/m² for 3 days (D-5~D-3) and cyclophosphamide at 750mg/m^2^ for 3 days (D-5~D-3). Antiemetic and symptomatic treatments can be given during chemotherapy. Except for contraindications, the general treatment is the same as that of other chemotherapies. If the neutrophil remains persistently < 1.0×10^9^/L after chemotherapy, granulocyte colony-stimulating factor could be applied until the neutrophil count is more than 1.5×10^9^/L. Antibiotics can be given to prevent infection if patients have neutropenia.

- - 1. **CAR T cells infusion**

CAR T cells will be infused two days after the end of chemotherapy. The CAR T cells are cryopreserved in insoluble cryomedia and will be administered as a single dose. The entire rewarm process should be within 10-15 minutes. During the entire infusion process, the patient's vital signs should be closely monitored. The oxygen saturation test should be performed 15 minutes before the infusion, at the end of the infusion, 15 minutes after the infusion, and until the patient's condition is stable. Patients will be given 325 to 650 mg of acetaminophen to prevent infusion-related reactions 30 to 60 minutes before infusion of CAR T cells. If fever occurs on the day of transfusion of CAR-T cells and lasts less than 24 hours without any other toxicities it is attributed to the transfusion of T-cell response.

- - 1. **Assessment and follow-up**

Assessment and follow-up were carried out at predefined time point after anti-BCMA/CD19 bispecific CAR T-cell infusion (day 0, 3, 7, 10, 14, 21, 28, 90±3, 120±7, 150±7, 180±7, 270±7, 360±7). After 1 year, regular follow-up will be continued by every six months. Participants will enter a at least three years long-term follow-up

If the subjects complete the D360 visit or are withdrawn from the group due to disease progression or intolerable toxicity during the treatment and visit, safety follow-up should be conducted. The overall safety observation period is three years after CAR T-cell infusion. Follow-up is performed every six months (either through in-hospital visits or telephone follow-ups, depending on the specific circumstances of the subjects). Follow-up continues until three years after CAR-T cell infusion, or until the subjects pass away, or until they initiate additional anti-tumor treatments. Safety data related to cell therapy are collected.

During the study period, for the safety of the subjects, if adverse events or abnormal laboratory test results occur in the subjects, the researchers may increase the number of follow-ups for the subjects as necessary, namely schedule field visits. The researchers are required to accurately record each scheduled field visit of the subjects in the unplanned follow-up section of the subjects' original documents and other materials.

1. **Termination of treatment and subject termination/withdrawal**
   1. **Subjects terminates the treatment**

The termination of treatment by the subjects does not mean the end of the research procedure. The subsequent research procedures should be completed in accordance with the provisions of the trial protocol.

For whatever reason, for the subjects who terminate the study treatment, the safety and efficacy data of the subjects should be obtained as much as possible. Under any circumstances, the reasons for withdrawal from the study should be recorded in the original documents of the subjects, and all evaluations at the end of treatment should be conducted when the subjects are willing and compliant.

- 1. **Subjects terminated/withdrew from the study**

Subjects have the right to withdraw from the study at any stage. Or, if a subject does not explicitly withdraw from the study but no longer receives medication or blood samples and is lost to follow-up, it also falls under "withdrawal" or "shedding".

The causes of detachment should be recorded, and all clinical evaluations and laboratory tests required at the end of the treatment plan visit should be completed as much as possible to ensure the safety of the subjects after detachment. On the premise of obtaining the consent of the subjects, their survival information should still be tracked and collected.

The exit criteria include but are not limited to the following categories:

(1) At any time during the study, disease progression occurs (after the subjects receive CAR-T cell infusion and reach the disease progression indicators stipulated in the protocol during the follow-up period, it is necessary for the researchers to determine and rule out the reactions related to therapy: such as abnormal indicators caused by CAR-T cell proliferation or aggregation in organs and tissues, etc.);

(2) During the research period, other treatments and those for the clinical research trial indications were received;

(3) Intolerable serious adverse events occurred and the researchers considered that the subjects were not suitable to continue the study;

(4) Voluntary withdrawal, that is, withdrawal of informed consent;

(5) The subject was lost to follow-up;

(6) The subjects had poor compliance and failed to strictly follow the research protocol.

(7) Pregnancy of female subjects;

(8) Technical difficulties occur during the modification and expansion of T cells, or the cell preparations fail to pass the quality inspection;

(9) The researchers believed that the subjects' conditions were not suitable for further participation in the study.

- 1. **Loss to Follow-up**

Loss to follow-up refers to a situation where, at different times, researchers repeatedly (more than three times) call the contact information provided by the subjects but still cannot reach them. The efforts made need to be recorded.

- 1. **Elimination Criteria**

(1) Those who have not used medication after inclusion or have no visit records at all;

(2) Non-prescribed combination medication, especially the use of drugs that have a significant impact on research results and affect the judgment of efficacy or safety;

(3) Other circumstances where the principal investigator determines that the trial protocol is seriously violated.

The excluded subjects should explain the reasons and their original documents should be retained for future reference.

1. **Evaluation of study**
   1. **Evaluation of therapeutic effect**
      1. **Disease assessment**

The disease-related examination results of the subjects at each predefined time point will be recorded as original data for each subject.

- - 1. **Evaluation of therapeutic effect**

The primary evaluation criteria include efficacy and safety.

Primary Indicators: Safety, including dose-limiting toxicity (DLT), maximum tolerated dose (MTD), and recommended dose.

Secondary indicators: Changes in the disease activity index of autoimmune diseases compared to baseline, as well as overall disease effectiveness. Researchers may determine the specific therapeutic effect by referring to the aforementioned indicators, along with the patient’s clinical manifestations and other relevant observations, integrating their professional judgment.

Secondary indicators for refractory myasthenia gravis: Changes in MG-ADL score, QMG score, Myasthenia Gravis Quality of Life 15-revised (MG-QoL-15r) score from day 0 (baseline) at each follow-up assessment up to 36 months. Clinical meaningful improvement is defined as a 2-point reduction in MG-ADL score or a 3-point reduction in QMG score.

Predefined exploratory indicators for refractory myasthenia gravis: changes in the proportion of participants achieving minimal manifestations or better based on MGFA Post-Intervention Status (MGFA-PIS), changes in anti-acetylcholine receptor antibody levels, and the proportion of those with glucocorticoid dosage tapered to 5 mg or less daily

- 1. **Evaluation of other research objectives**
     1. **Safety Assessment**

Safety evaluations will be conducted during the screening period, throughout the research process, and after the completion of the study. Participants who withdraw prematurely must undergo a safety evaluation prior to discontinuation.

Safety Evaluation Indicators: adverse events, cytokine release syndrome (CRS), vital signs, general physical examination, ECOG score, laboratory tests, electrocardiogram, echocardiography, etc.

1) Adverse Events: Definitions, assessments, and follow-up procedures are detailed in Section 8.3.

2) Cytokine Release Syndrome: including levels of cytokines, ferritin, and CRP.

3) Vital Signs: Respiratory rate, pulse, blood pressure, blood oxygen saturation, and body temperature will be monitored according to the study schedule.

4) General Physical Examination: A comprehensive physical examination will be performed in accordance with the procedures, covering skin, mucous membranes, lymph nodes, head, neck, chest, abdomen, spine/limbs, and neurological examinations.

5) ECOG Score.

- 1. **Adverse Events and Serious Adverse Events**
     1. **Definition of Adverse Events Adverse events (AEs)**

Definition of AEs are defined as any unfavorable medical occurrences that manifest following the administration of the investigational drug to study subjects. These events may present as symptoms, signs, diseases, or abnormal laboratory findings, although a causal relationship with the investigational drug is not necessarily implied.

- - 1. **Definition of Serious Adverse Events Serious adverse events (SAEs)**

Definition of SAEs refer to adverse events that meet any one of the following criteria: 1) Result in death; 2) Are life-threatening; 3) Require hospitalization or prolongation of existing hospitalization; 4) Lead to persistent or significant disability or incapacity; 5) Cause congenital anomalies or birth defects; 6) Constitute other significant medical events. Events that do not immediately result in death, life-threatening conditions, or hospitalization but are deemed by appropriate medical judgment to place the subject at risk of such outcomes or require intervention to prevent them are also generally classified as serious adverse events.

- - 1. **Recording and description of AEs/SAEs**

All AEs and SAEs observed or reported by study participants during the course of the study were accurately documented by the research team in the original medical records. The following principles should be followed when recording and describing adverse events: (1) Completeness: The documentation in the original medical records should include, but not be limited to, basic trial and participant information, investigational drug usage, occurrence of adverse events, treatment measures taken in response to adverse events, actions taken regarding the investigational drug, outcomes of adverse events, causality assessment and its basis, as well as concomitant medications. (2) Readability: Medical terminology should be expressed in full form whenever possible, avoiding abbreviations to minimize ambiguity.

- - 1. **The severity of AEs/SAEs**

Researchers will utilize the NCI CTCAE (Version 5.0) to evaluate adverse events and classify their severity. In cases where the severity of a specific adverse event is not explicitly defined in the guidelines, investigators may determine its grade based on the standard definitions for Grades 1 to 5, in conjunction with clinical judgment. Standard classification of adverse events:

Grade 1: Mild; asymptomatic or mild symptoms; intervention not indicated.

Grade 2: Moderate; requiring minor, local, or non-invasive intervention; limitations in instrumental activities of daily living appropriate to age.

Grade 3: Severe or medically significant but not immediately life-threatening; may require hospitalization or prolong existing hospitalization; limitations in self-care activities of daily living.

Grade 4: Life-threatening; urgent intervention required.

Grade 5: Death related to the adverse event.

- - 1. **Judgment of AEs/SAEs**

According to the five criteria for adverse event analysis, the relationship between an adverse event and the investigational drug is assessed using five categories: “definitely related,” “very likely related,” “possibly related,” “possibly unrelated,” and “definitely unrelated.” When the causal relationship falls under "definitely related," "very likely related," or "possibly related," the event is considered to be associated with the investigational drug.

Definitely related: The event corresponds to a known reaction type of the suspected drug, follows a reasonable temporal sequence after administration, diminishes or resolves upon dose reduction or drug discontinuation, and recurs upon re-administration of the drug.

Very likely related: The event corresponds to a known reaction type of the suspected drug and follows a reasonable temporal sequence after administration. It improves or resolves after dose reduction or discontinuation; however, the subject's clinical condition or other factors may also have contributed to the occurrence of the event.

Possibly related: Conforming to the known reaction type of the suspected drug, conforming to the reasonable time sequence after medication, and the adverse event alleviated or not obvious after dose reduction or drug withdrawal, but the clinical state of the subject or other reasons can explain the event.

Possibly unrelated: It does not quite conform to the known reaction type of the suspected drug, does not quite conform to the reasonable time sequence after medication, and the clinical state of the subject or other reasons may also cause this event.

Definitely unrelated: It does not conform to the known reaction type of the suspected drug, does not conform to the reasonable time sequence after medication, and the clinical state of the subject or other reasons can also explain this reaction. After excluding clinical symptoms or other reasons, the event alleviates or disappears.

- - 1. **Follow-up of adverse events**

Adverse events should be observed and documented from the time of informed consent form signing through to the conclusion of the safety follow-up period. All reported adverse events must be followed until resolution, stabilization of the condition, subject loss to follow-up as determined by the investigator, or until a satisfactory explanation for the event is obtained. During the safety follow-up phase, only adverse events deemed related to the cell therapy administered in this study were recorded.

The end time of adverse events should be considered as the recovery of the adverse events, the stabilization of the condition, or a reasonable explanation. If, at the time of the subject's death, no end time was collected and the adverse event that was not the direct cause of death persisted, the end time of the adverse event should be left blank and the status should be marked as “persistent”. If the adverse event is determined to be the direct or main cause of “death”, the end time is the time of the subject's death.

- 1. **Safety report**

Suspected and unexpected serious adverse reactions are those whose nature and severity of clinical manifestations go beyond the existing data information, such as that in the investigational drug investigator's manual or product characteristic summary. Researchers should report suspected and unexpected serious adverse reactions to research partners, ethics committees, and relevant regulatory authorities in a timely manner.

| Procedures | Screening | Preparation for CAR T cells | Lymphodepleting Chemotherapy | | | CAR T- cell infusion | Assessment and follow-up | | | | | | | | | | | | | | | | Safety Assessment^u^ |
| --- | --- | --- | --- | --- | --- | --- | --- | --- | --- | --- | --- | --- | --- | --- | --- | --- | --- | --- | --- | --- | --- | --- | --- |
| Schedule | D-48~  D-28 | D-28 ~  D-1 | D-5 | D-4 | D-3 | D0 | D3 | D7 | D10 | D14  ±3 | D21  ±3 | D28  ±3 | D90  ±3 | D120  ±3 | D150  ±3 | D180  ±7 | D270  ±14 | D360  ±14 | M18 | M24 | M30 | M36 |  |
| Informed consent^a^ | X |  |  |  |  |  |  |  |  |  |  |  |  |  |  |  |  |  |  |  |  |  |  |
| Inclusion/Exclusion Criteria | X |  |  |  |  |  |  |  |  |  |  |  |  |  |  |  |  |  |  |  |  |  |  |
| Demographic data^b^ | X |  |  |  |  |  |  |  |  |  |  |  |  |  |  |  |  |  |  |  |  |  |  |
| Medical history^c^ | X |  |  |  |  |  |  |  |  |  |  |  |  |  |  |  |  |  |  |  |  |  |  |
| Height/Weight^e^ | X |  | X |  |  | X |  |  |  |  |  |  |  |  |  |  |  |  |  |  |  |  |  |
| Vital signs^f^ | X |  |  |  | X | X | X | X | X | X | X | X | X | X | X | X | X | X | X | X | X | X | X |
| physical examination^g^ | X |  |  |  | X | X | X | X | X | X | X | X | X | X | X | X | X | X | X | X | X | X | X |
| ECOG | X |  |  |  | X |  |  |  |  |  |  | X | X | X | X | X | X | X | X | X | X | X | X |
| Blood routine/biochemistry test^h^ | X |  |  |  | X |  | X | X | X | X | X | X | X | X | X | X | X | X | X | X | X | X | X |
| Urine routine test^i^ | X |  |  |  | X |  | X | X | X | X | X | X | X | X | X | X | X | X | X | X | X | X | X |
| routine stool and occult blood test^j^ | X |  | A routine stool test and occult blood test must be conducted once each 2 days before CAR-T cell infusion and within 7 days after infusion. Other tests are carried out based on the judgment of the researchers. | | | | | | | | | | | | | | | | | | | | X |
| Pregnancy test^k^ | X |  | X |  |  |  |  |  |  |  |  |  |  |  |  |  |  | X |  |  |  |  | X |
| Infectious disease testing^l^ | X |  |  |  |  |  |  |  |  |  |  |  |  |  |  | X |  | X |  |  |  |  | X |
| Cytokines | X |  |  |  | X |  | X | X | X | X | X | X | X | X | X | X | X | X | X | X | X | X |  |
| Ferritin/CRP | X |  |  |  | X |  | X | X | X | X | X | X | X | X | X | X | X | X | X | X | X | X |  |
| Ultrasonic cardiogram | X |  |  |  |  |  |  |  |  |  |  | X |  |  |  |  |  | X |  | X |  | X | X |
| Electrocardiography^m^ | X |  |  |  | X |  |  | X |  | X | X | X |  |  |  | X |  | X | X | X | X | X | X |
| Efficacy evaluation^n^ | X |  |  |  |  |  | X |  |  | X |  | X | X | X | X | X | X | X | X | X | X | X | X |
| Immunoglobulin^o^ | X |  |  |  |  | X |  | X |  | X | X | X | X | X | X | X | X | X | X | X | X | X | X |
| Imaging assessment^p^ | X |  |  |  |  | The decision to conduct the examination is made by the researcher based on the specific characteristics of the subjects, and the selection of specific detection methods is also determined by the researcher. | | | | | | | | | | | | | | | | |  |
| Other tests related to autoimmune diseases | X |  |  |  |  | The decision to conduct the examination is made by the researcher based on the specific characteristics of the subjects, and the selection of specific detection methods is also determined by the researcher. | | | | | | | | | | | | | | | | |  |
| Cyclophosphamide |  |  | X | X | X |  |  |  |  |  |  |  |  |  |  |  |  |  |  |  |  |  |  |
| Fludarabine |  |  | X | X | X |  |  |  |  |  |  |  |  |  |  |  |  |  |  |  |  |  |  |
| CAR T-cell infusion |  |  |  |  |  | X |  |  |  |  |  |  |  |  |  |  |  |  |  |  |  |  |  |
| Immunogenic blood collection^q^ |  |  |  |  |  | X |  |  |  | X | X | X | X | X | X | X | X | X | X | X | X | X | X |
| Adverse event^r^ | X | X |  | X | X | X | X | X | X | X | X | X | X | X | X | X | X | X | X | X | X | X | X |
| Concomitant treatment^s^ | X | X |  | X | X | X | X | X | X | X | X | X | X | X | X | X | X | X | X | X | X | X | X |
| Dose-limiting toxicity^t^ |  |  |  |  |  | X | | | | | | |  |  |  |  |  |  |  |  |  |  |  |

Items related to the safety of vital signs, general physical examination, cytokines (IL-2, IL-4, IL-6, IL-8, IL-10, TNF-α, IFN-γ), etc. Within one month after cell infusion, the examination density can be increased by the researchers according to the conditions of the subjects.

The results of echocardiography and electrocardiogram within 14 days before signing the informed consent form during the screening period;

a) Informed consent must be obtained before conducting any specific steps of the research;

b) Demographic data include date of birth, gender, ethnicity, age, nationality, etc.

c) Medical history includes current illness history (especially diagnostic information for diseases), previous disease history, medication history, allergy history, alcohol consumption history, drug abuse history, blood transfusion history, clinical trial history, previous surgical history, etc.

d) Consistency assessment of the condition: Assessment of the subjects being diagnosed with autoimmune diseases;

e) Height/weight: Measured during the screening period, before the first treatment with cyclophosphamide + fludarabine, and before the reinfusion of CAR-T products;

f) Vital signs include: respiratory rate, pulse, blood pressure, blood oxygen saturation and body temperature.

g) Physical examination: including skin, mucous membranes, lymph nodes, head, neck, chest, abdomen, spine/limbs, nervous system examination, etc.

h) Blood routine/biochemical tests include: red blood cell count (RBC), hemoglobin (HGB), platelet count (PLT), white blood cell count (WBC), absolute neutrophil count (Neut#), absolute lymphocyte count (LYMPH#), albumin (ALB), alanine aminotransferase (ALT), aspartate aminotransferase (AST), alkaline phosphatase (ALP), glutamyl transpeptidase (GGT), total bilirubin (TBIL), direct bilirubin (DBIL), Urea (Urea) or blood urea nitrogen (BUN), creatinine (CREA), creatine kinase (CK) Lactate dehydrogenase (LDH), triglyceride (TG), cholesterol (CHOL), high-density lipoprotein cholesterol (HDL), low-density lipoprotein cholesterol (LDL), blood glucose (GLU), electrolytes (potassium, sodium, chlorine, calcium, magnesium, phosphorus), procalcitonin (PCT), erythrocyte sedimentation rate (ESR), etc.

i) Urine routine includes: urine glucose (UGLU), ketone body qualitative test (KET), urine bilirubin qualitative test (BIL), urine bilirubin qualitative test (UBG), urine protein qualitative test (PRO), PH, occult blood (BLD), red blood cells (RBC), white blood cells (WBC), etc.

j) Routine stool and occult blood test: Whether to conduct the test is determined by the researcher based on the specific conditions of the subjects. However, routine stool test + occult blood test must be conducted once each 2 days before the infusion of anti-BCMA-CD19 CAR-T cells and within 7 days after the infusion.

k) Pregnancy examination: Only for women of childbearing age. Blood pregnancy examination should be conducted during the screening period and before the first treatment with cyclophosphamide + fludarabine, as well as during the group exit visit.

l) Infectious disease detection includes: hepatitis B two pairs and a half (five items), hepatitis B virus DNA (HBV-DNA), hepatitis C virus (HCV) antibody, hepatitis C virus RNA (HCV-RNA), human immunodeficiency virus antibody (HIV (1+2) Ab);

m) Electrocardiogram examination: All electrocardiograms should be performed 3 minutes after being in a supine or semi-supine position.

n) Efficacy evaluation: Regular evaluation during the screening period and after infusion;

o) Immunoglobulins: IgG, IgA, IgM, complement C3, C4;

p) Imaging assessment: The researcher will determine whether to conduct the examination based on the specific conditions of the subjects. The specific detection methods will be decided by the researcher.

q) Immunogenic blood collection: About 5mL of venous blood was collected 30 minutes before the infusion of anti-BCMA-CD19 CAR-T cells (D0), and at D7, D14, D28, D60, D90, D120, D150, D180, D270, D360 after the infusion and during safety follow-ups (such as hospital visits) to measure the antibodies of anti-CAR-T positive cells.

r) Adverse events (AE) : Adverse events need to be observed and recorded from the signing of the informed consent form until the end of the safety follow-up. The adverse events that have occurred should be followed up until recovery, stable status, loss to follow-up of the subject as judged by the investigator, or other explanations for the event can be provided. During the safety follow-up stage, only adverse events related to cell therapy were recorded;

s) Concomitant treatment: Combined medication should be recorded from the signing of the informed consent form to the end of the safety follow-up. During the safety follow-up stage, only the combined medication and other anti-tumor treatments (if any) related to adverse events of cell therapy were recorded;

t) Dose-limiting toxicity assessment was conducted only during the dose escalation stage, and the DLT observation period was within 28 days after cell infusion;

u) Safety assessment was conducted if the participants completed the D360 visit or were removed from the group due to disease progression or intolerable toxicity during the treatment and visit. The overall safety observation period was 3 years after CAR-T cell infusion. Follow-up was conducted every 3 months (either by in-hospital visits or telephone follow-ups based on the specific conditions of the subjects), and the follow-up was carried out until 3 years after CAR-T cell infusion, or until the subjects died, or until the subjects began additional disease treatment for the indications involved in the clinical trial. Collect the safety data related to the cell therapy of this research project.

# References

1. Shi M, Wang J, Huang H, et al. Bispecific CAR T cell therapy targeting BCMA and CD19 in relapsed/refractory multiple myeloma: a phase I/II trial. Nat Commun. 2024 Apr 20;15(1):3371.
2. Zhang Y, Liu D, Zhang Z, et al. Bispecific BCMA/CD19 targeted CAR-T cell therapy forces sustained disappearance of symptoms and anti-acetylcholine receptor antibodies in refractory myasthenia gravis: a case report. J Neurol. 2024 Jul;271(7):4655-4659.
3. Iwata S, Saito K, Hirata S, et al. Efficacy and safety of anti-CD20 antibody rituximab for patients with refractory systemic lupus erythematosus. Lupus. 2018; 27(5): 802-811.
4. Henry J, Gottenberg JE, Rouanet S, et al. Doses of rituximab for retreatment in rheumatoid arthritis: influence on maintenance and risk of serious infection. Rheumatology (Oxford). 2018; 57(3): 538-547.
5. McAdoo SP, Medjeral-Thomas N, Gopaluni S, et al. Long-term follow-up of a combined rituximab and cyclophosphamide regimen in renal anti-neutrophil cytoplasm antibody-associated vasculitis. Nephrol Dial Transplant. 2018; 33(5): 899.
6. Gross G, Waks T, Eshhar Z. Expression of immunoglobulin-T-cell receptor chimeric molecules as functional receptors with antibody-type specificity. Proc Natl Acad Sci USA. 1989; 86(24): 10024-8.
7. Park JH, Rivière I, Gonen M, et al. Long-Term Follow-up of CD19 CAR Therapy in Acute Lymphoblastic Leukemia. N Engl J Med. 2018; 378(5): 449-459.
8. Neelapu SS, Locke FL, Bartlett NL, et al. Axicabtagene Ciloleucel CAR T-Cell Therapy in Refractory Large B-Cell Lymphoma. N Engl J Med. 2017; 377(26): 2531-2544.
9. Lee DW, Kochenderfer JN, Stetler-Stevenson M, et al. T cells expressing CD19 chimeric antigen receptors for acute lymphoblastic leukaemia in children and young adults: a phase 1 dose-escalation trial. Lancet. 2015; 385(9967): 517-28.
10. Kochenderfer JN, Dudley ME, Kassim SH, et al. Chemotherapy-refractory diffuse large B-cell lymphoma and indolent B-cell malignancies can be effectively treated with autologous T cells expressing an anti-CD19 chimeric antigen receptor. J Clin Oncol. 2015; 33(6): 540-9.
11. Maude SL, Laetsch TW, Buechner J, et al. Tisagenlecleucel in Children and Young Adults with B-Cell Lymphoblastic Leukemia. N Engl J Med. 2018; 378(5): 439-448.
12. Raje N, Berdeja J, Lin Y, et al. Anti-BCMA CAR T-Cell Therapy bb2121 in Relapsed or Refractory Multiple Myeloma. N Engl J Med. 2019; 380(18): 1726-1737.
13. Munshi NC, Anderson LD Jr, Shah N,et al. Idecabtagene Vicleucel in Relapsed and Refractory Multiple Myeloma. N Engl J Med. 2021; 384(8): 705-716.
14. Zhao WH, Liu J, Wang BY, et al. A phase 1, open-label study of LCAR-B38M, a chimeric antigen receptor T cell therapy directed against B cell maturation antigen, in patients with relapsed or refractory multiple myeloma. J Hematol Oncol. 2018; 11(1):141.
15. Xu J, Chen LJ, Yang SS, et al. Exploratory trial of a biepitopic CAR T-targeting B cell maturation antigen in relapsed/refractory multiple myeloma. Proc Natl Acad Sci U S A. 2019; 116(19): 9543-9551.
16. Berdeja JG, Madduri D, Usmani SZ, et al. Ciltacabtagene autoleucel, a B-cell maturation antigen-directed chimeric antigen receptor T-cell therapy in patients with relapsed or refractory multiple myeloma (CARTITUDE-1): a phase 1b/2 open-label study. Lancet. 2021; 398(10297): 314-324.
17. Shi M, Wang J, Huang H, et al. Bispecific CAR T cell therapy targeting BCMA and CD19 in relapsed/refractory multiple myeloma: a phase I/II trial. Nat Commun. 2024 Apr 20;15(1):3371.

# Appendix

**Appendix 1**

Primary research institutions and collaborative research partners

| Study name | Safety and Efficacy of Anti-BCMA/CD19 Bispecific CAR T-Cell Therapy in the Treatment of Autoimmune Diseases |
| --- | --- |
| Responsible Unit and Individual | Jiang Cao  The Affiliated Hospital of Xuzhou Medical University  No. 99 Huaihai West Road, Quanshan District, Xuzhou, Jiangsu, China  Telephone number: 0086-13852432263  E-mail address：zimu05067@163.com |

**Appendix 2**

ECOG Performance Status Scale

| Grade | Performance status |
| --- | --- |
| 0 | Fully active, able to carry on all pre-disease performance without restriction |
| 1 | Restricted in physically strenuous activity but ambulatory and able to carry out work of a light or sedentary nature, e.g., light house work, office work |
| 2 | Ambulatory and capable of all selfcare but unable to carry out any work activities; up and about more than 50% of waking hours |
| 3 | Capable of only limited selfcare, confined to bed or chair more than 50% of waking hours |
| 4 | Completely disabled; cannot carry on any selfcare, totally confined to bed or chair |
| 5 | Dead |

ECOG=Eastern Cooperative Oncology Group

**Appendix 3**

**Management Approaches for Adverse Events**

All adverse event management should initially involve consultation with specialist physicians from the relevant departments. Following the integration of their professional opinions, appropriate interventions should then be implemented. The handling measures outlined below are provided solely for reference purposes.

1. **Adverse event associated with infusion of anti-BCMA/CD19 CAR T-cell**

The adverse event associated with CAR-T cell therapy primarily include cytokine release syndrome, neurotoxicity, macrophage activation syndrome, B-cell regeneration disorders, and hypogammaglobulinemia. Following CAR-T cell infusion, it is essential to closely monitor the subjects for symptoms such as cough, low-grade fever, fatigue, myalgia, chills, diaphoresis, anorexia, nausea, and diarrhea, as well as any signs related to respiratory or cardiovascular dysfunction. During the first two weeks after initial cell administration, particular attention should be given to the potential occurrence of tumor lysis syndrome. Key monitoring parameters include serum electrolytes, phosphorus, calcium, uric acid, creatinine, and lactate dehydrogenase levels. Toxicity assessments were carried out in accordance with the predefined study protocol.

- 1. **Cytokine release syndrome (CRS)**

The diagnostic criteria for cytokine release syndrome are as follows: 1) Fever persists for at least three consecutive days; 2) The peak concentration of at least two different cytokines increases by no less than 75-fold, or the peak concentration of a single cytokine increases by at least 250-fold; 3) At least one clinical manifestation of toxicity is present, such as hypotension (requiring vasopressor support), hypoxia (with blood oxygen saturation below 90%), or neurological symptoms (including altered mental status, lethargy, or seizures). The CRS grade system were listed as Table 1.

Management: If the patient presents with fever, antipyretic and symptomatic supportive treatment may be administered. For patients with a CRP level ≥200 mg/L, transfer to the intensive care unit for specialized monitoring and management is recommended. In the presence of clinical manifestations such as hypotension, hypoxemia, or organ toxicity, consideration should be given to initiating treatment with tocilizumab. Should the aforementioned symptoms persist following the second dose of tocilizumab, corticosteroids such as intravenous dexamethasone should be initiated.

**Table 1. CRS grade system**

| Parameter | Grade 1 | Grade 2 | Grade 3 | Grade 4 |
| --- | --- | --- | --- | --- |
| Fever | Temperature ≥38°C | | | |
| Hypotension | None | Not requiring  vasopressors | Requiring one vasopressor with or without vasopressin | Requiring multiple vasopressors (excluding  vasopressin) |
| Hypoxia | None | Requiring low flow rate (≤ 6L/min or cross-flow)  Inhale oxygen through nasal cannula | Requiring high flow rate (> 6L/min)  Inhale oxygen through nasal cannula, mask or Venturi mask | Requiring positive pressure ventilation (continuos positive airway pressure, Bi-level positive airway pressure, intubation or mechanical ventilation) |

- 1. **Neurological toxicity**

Neurotoxic toxicity may manifest as confusion, delirium, expressive aphasia, lethargy, myoclonus, and epileptic seizures. The underlying pathophysiological mechanisms of these symptoms are not yet fully understood and may be related to elevated cytokine levels within the central nervous system.

The neurological function of patients can be assessed using the CAR-T-cell-therapy-associated Toxicity (CARTOX)-10 scale, which assigns a total score of 10 to individuals with normal cognitive function. The assessment includes orientation testing regarding time (year and month) and place (city and hospital name), as well as knowledge of the national leader’s name, for a total of 5 points. Additionally, patients are asked to name three objects—such as a clock, a pen, and a button—for up to 3 points. They are also required to write a complete sentence, such as “My name is XXX” (1 point), and perform serial subtraction from 100 in decrements of 10 (1 point). The neurological toxicity grade system were listed as Table 2.

**Table 2. Neurological toxicity grade system**

| Grade | Performance |
| --- | --- |
| 1 | The CARTOX-10 score was 7 to 9 points (mild impairment), with no increase in intracranial pressure and no epileptic seizures |
| 2 | The CARTOX-10 score was 3 to 6 points (moderately impaired), with no increase in intracranial pressure and no epileptic seizures |
| 3 | CARTOX-10 score: 0-2 points (severe impairment), grade 1-2 optic disc edema, or intracranial pressure <20 mmHg. Partial seizures of epilepsy, or non-convulsive seizures that respond effectively to benzodiazepine treatment |
| 4 | In a critical state and/or sluggish response that cannot be evaluated, grade 3 to 5 papillary edema or intracranial pressure ≥20 mmHg or cerebral edema; Generalized epileptic seizures, or convulsive/non-convulsive status epilepticus, or newly emerged limb weakness |

- 1. **Macrophage Activation Syndrome and Hemophagocytic Lymphohistiocytosis**

In patients with CRS who present significantly elevated levels of ferritin, C-reactive protein (CRP), and soluble interleukin-2 receptor (sIL-2R), macrophage activation syndrome (MAS) or hemophagocytic lymphohistiocytosis (HLH) may develop. The primary clinical manifestations include fever, splenomegaly, pancytopenia, hypertriglyceridemia, hypofibrinogenemia, elevated serum ferritin, and evidence of hemophagocytosis detectable in bone marrow, spleen, or lymph node biopsies.

Management: Close monitoring and timely referral to the established clinical guidelines for MAS/HLH are recommended. For patients with severe CRS complicated by MAS/HLH, combination therapy with the IL-6 receptor antagonist tocilizumab and corticosteroids is indicated.

- 1. **B-cell aplasia and hypogammaglobulinemia**

Definition of B-cell aplasia was as follows: (1) CD19+ B cells were undetected in the bone marrow and peripheral blood B lymphocyte count < 0.01×10^9^/L at least 2 weeks and more than twice; or (2) CD19^+^ B cells in peripheral blood constitute less than 1% of the total lymphocyte population.

Definition of hypogammaglobulinemia: serum IgG < 4.0g/L.

Management: Intravenous immunoglobulin infusion is recommended. The recommended dosage is 0.4g–0.6g/kg per month. The trough IgG concentration (measured after completion of a treatment cycle) should be monitored to ensure that it remains at or above the lower limit of the normal reference range.

- 1. **Contamination of CAR T cells**

The likelihood of sepsis or systemic bacterial infection during the infusion of CAR T cells is extremely low. Nevertheless, if subjects experience persistent fever reactions following CAR T-cell infusion, after ruling out fever reactions caused by other adverse reactions such as cytokine release syndrome, the possibility of bacterial contamination should be taken into account.

Management: Appropriate bacterial cultures should be promptly performed, and corresponding antibacterial drugs should be administered. If there is a suspicion of anti-BCMA/CD19 CAR T cell contamination, the product should be retested for sterility.

1. **Adverse event associated with lymphodepleting chemotherapy**

To improve the survival rate of anti-BCMA/CD19 CAR T cells, all participants must undergo lymphodepleting chemotherapy, which consists of cyclophosphamide and fludarabine, before the cell infusion. Cyclophosphamide can lead to adverse effects such as myelosuppression, alopecia, gastrointestinal issues, stomatitis, and cystitis. Fludarabine primarily induces dose-dependent myelosuppression, including neutropenia and anemia.

Management: Since this is a short-term treatment regimen (administered over three days), the likelihood of inducing severe adverse effects is relatively low. In the event of any of the aforementioned side effects occurring, close monitoring should be the primary course of action. Should serious adverse effects arise or the patient's condition change significantly, the physician will implement appropriate symptomatic treatment based on clinical judgment and individual patient status.

1. **Management of tocilizumab**

Acute cytokine release syndrome during CAR T-cell therapy often necessitates intensive care management. In cases where cytokine release syndrome cannot be adequately controlled with corticosteroids, anti-cytokine agents such as tocilizumab have shown efficacy without causing non-specific immunosuppression. Currently, this approach is regarded as a standard therapeutic strategy for managing cytokine release syndrome in CAR T-cell therapy. The most commonly observed adverse effects (with an incidence rate of approximately 5%) include upper respiratory tract infections, nasopharyngitis, headache, hypertension, and elevated alanine aminotransferase levels.

Warnings and Precautions: Serious and potentially fatal infections, including those caused by bacterial, mycobacterial, fungal, viral, protozoal, or other opportunistic pathogens, have been reported in patients receiving immunosuppressants. Common serious infections associated with tocilizumab encompass pneumonia, urinary tract infections, cellulitis, herpes zoster, enteritis, diverticulitis, sepsis, and bacterial arthritis. Additionally, cases of opportunistic infections such as tuberculosis, cryptococcosis, Aspergillus, Candida, and Pneumocystis jirovecii pneumonia have been documented. Other rare but severe infections not identified in clinical trials may also occur, such as histoplasmosis, coccidioidomycosis, and listeriosis. Patients with systemic rather than localized disease, who are also receiving immunosuppressive agents like methotrexate or glucocorticoids, may be at an increased risk of infection. Consequently, tocilizumab should not be administered to patients with active infections, including localized infections.

**Appendix 4**

**Quantitative Myasthenia Gravis scale (QMG)**

|  | Category | Normal (0) | Mild (1) | Moderate (2) | Severe (3) | Score |
| --- | --- | --- | --- | --- | --- | --- |
| Ocular Muscles | 1. Diplopia on lateral gaze (seconds) | ≥61 | 11–60 | 1–10 | Spontaneous |  |
|  | 1. Ptosis on upward gaze (seconds) | ≥61 | 11–60 | 1–10 | Spontaneous |  |
|  | 1. Eyelid closure | Normal | Closes with partial resistance | Cannot close against resistance | Unable to close |  |
| Bulbar Muscles | 1. Swallowing 100mL water | Normal | Mild choking | Severe choking/nasal regurgitation | Unable to swallow |  |
|  | 1. Counting 1–50 (dysarthria) | No dysarthria | 30–49 numbers | 10–29 numbers | 0–9 numbers |  |
| Proximal Arm Muscles | 1. Right arm abduction 90° (seconds) | ≥240 | 90–239 | 10–89 | 0–9 |  |
|  | 1. Left arm abduction 90° (seconds) | ≥240 | 90–239 | 10–89 | 0–9 |  |
| Respiratory Muscles | 1. Vital capacity (% predicted) | ≥80% | 65–79% | 50–64% | <50% |  |
| Distal Arm Muscles | 1. Right Grip, kg |  |  |  |  |  |
|  | - Male | ≥45 | 15–44 | 5–14 | 0–4 |  |
|  | - Female | ≥30 | 10–29 | 5–9 | 0–4 |  |
|  | 1. Left Grip, kg |  |  |  |  |  |
|  | - Male | ≥35 | 15–34 | 5–14 | 0–4 |  |
|  | - Female | ≥25 | 10–24 | 5–9 | 0–4 |  |
| Neck Muscles | 1. Head lift 45° (supine, seconds) | ≥120 | 30–119 | 1–29 | 0 |  |
| Leg Muscles | 1. Right leg lift 45° (supine, seconds) | ≥100 | 31–99 | 1–30 | 0 |  |
|  | 1. Left leg lift 45° (supine, seconds) | ≥100 | 31–99 | 1–30 | 0 |  |
| Total QMG Score | | | | | |  |

**Appendix 5**

**Myasthenia Gravis Activities of Daily Living Scale (MG-ADL)**

| Assessment Items | Normal (0) | Mild (1) | Moderate (2) | Severe (3) | Score |
| --- | --- | --- | --- | --- | --- |
| Speech | Normal | Unclear speech or nasal voice, intermittent | Consistently unclear but understandable | Unintelligible speech |  |
| Chewing | Normal | Difficulty with hard foods | Difficulty with soft foods (diet modification needed) | Requires feeding tube |  |
| Swallowing | Normal | Occasional choking | Frequent choking (diet modification needed) | Requires feeding tube |  |
| Breathing | Normal | Shortness of breath on exertion | Shortness of breath at rest | Requires ventilator |  |
| Brushing teeth/Combing hair | Normal | Needs assistance from other hand (no breaks) | Needs intermittent breaks | Unable to perform |  |
| Rising from a chair | Normal | Mild (occasionally uses hands) | Moderate (always uses hands) | Severe (needs assistance) |  |
| Diplopia (Double vision) | Normal | Occurs intermittently (not daily) | Daily (not constant) | Constant |  |
| Ptosis (Eyelid drooping) | Normal | Occurs intermittently (not daily) | Daily (not constant) | Constant |  |
| Total MG-ADL Score | | | | |  |

**Appendix 6**

**Myasthenia Gravis Quality of Life 15-revised (MG-QOL15r)**

| Please describe how true each statement has been for you (in the past few weeks) | Not at all | A little | Very much |
| --- | --- | --- | --- |
|  | 0 | 1 | 2 |
| 1. I feel frustrated because of my MG. |  |  |  |
| 1. Because of MG, I have vision problems (e.g., double vision). |  |  |  |
| 1. Because of MG, I have difficulty eating. |  |  |  |
| 1. Because of MG, I have to limit my social activities. |  |  |  |
| 1. MG limits my ability to enjoy hobbies and leisure activities. |  |  |  |
| 1. Because of MG, I have trouble meeting my family’s needs. |  |  |  |
| 1. I have to plan my life around MG. |  |  |  |
| 1. I feel troubled by MG restricting my work (including household tasks). |  |  |  |
| 1. Because of MG, I have difficulty speaking. |  |  |  |
| 1. Because of MG, I’ve lost some independence (e.g., driving, shopping, running errands). |  |  |  |
| 1. I feel upset about having MG. |  |  |  |
| 1. Because of MG, I have difficulty walking. |  |  |  |
| 1. Because of MG, I find it hard to be active in public places. |  |  |  |
| 1. I feel overwhelmed by MG |  |  |  |
| 1. Because of MG, I have difficulty with personal hygiene. |  |  |  |
| Total MG-QOL15r score: |  | | |
