# Supplementary material for: Anti-BCMA/CD19 CAR T cell therapy in patients with refractory generalized myasthenia gravis: a single-arm, phase 1 trial
Source: eClinicalMedicine. 2025 Oct 31;90:103621. doi: 10.1016/j.eclinm.2025.103621 (PMC12615342; doi:10.1016/j.eclinm.2025.103621)
Supplement: Supplementary Material 2 [file mmc2.docx]

**Anti-BCMA/CD19 CAR T cells therapy in patients with refractory generalized myasthenia gravis: a single-arm, open-label, phase 1 trial**

**Table of Contents**

[Supplement Tables](#_Toc6747)

[Table S1. Detailed Demographics and baseline characteristics 2](#_Toc24545)

[Table S2. Detailed treatment before CAR T-cell infusion 3](#_Toc26255)

[Table S3. Percentage of BCMA/CD19 CAR positive T cells 4](#_Toc26355)

[Table S4. Summary of cytokine release syndrome 5](#_Toc6637)

[Table S5. Summary of grade 3-4 hematological toxicities 6](#_Toc20540)

[Table S6. Changes in serum cytokine levels within 28 days after CAR T-cell infusion of all participants 7](#_Toc15504)

[Table S7. Changes in daily dose of prednisone and pyridostigmine after CAR T-cell infusion. 8](#_Toc4866)

[Table S8. Changes in proportion of peripheral CD19+ B cells after CAR T-cell infusion. 9](#_Toc8655)

[Table S9. Changes in anti-AChR antibodies titer after CAR T-cell infusion. 10](#_Toc20905)

[Supplement Figures](#_Toc13641)

[Figure S1. Structure of the anti-BCMA/CD19 bispecific CAR T cells 11](#_Toc22804)

[Figure S2. Changes in IL-6 and hs-CRP after CAR T-cell in all participants 12](#_Toc18722)

[Figure S3. Changes in serum IgG, IgM, and IgA after CAR T-cell infusion in different dose 13](#_Toc20571)

[Figure S4. Amplification of anti-BCMA/CD19 bispecific CAR T cells 14](#_Toc14195)

[Figure S5. Changes in the proportions of CD3](#_Toc17168)^[+](#_Toc17168)^ [T, CD4](#_Toc17168)^[+](#_Toc17168)^ [T, CD8](#_Toc17168)^[+](#_Toc17168)^ [T, and NK cells among total lymphocytes after CAR T-cell infusion 15](#_Toc17168)

[Figure S6. Changes in the proportion of participants with different MG-ADL score interval 16](#_Toc30719)

[Figure S7. Changes in the proportion of participants according to MGFA classification 17](#_Toc17044)

# Supplement Tables

## Table S1. Detailed Demographics and baseline characteristics

| No. | Age | Sex | Weight (kg) | BMI (kg/m^2^) | Age at onset (years) | Duration (months) | Myasthenic crisis | Thymoma | Thymectomy | Time since thymectomy (months) | Baseline disease severity | | | | AChR antibody (nmol/L)^*^ |
| --- | --- | --- | --- | --- | --- | --- | --- | --- | --- | --- | --- | --- | --- | --- | --- |
|  |  |  |  |  |  |  |  |  |  |  | MGFA classification | QMG score | MG-ADL  score | MG-QOL15r score |  |
| 1 | 64 | M | 76 | 26.3 | 57 | 81 | Y | Y | Y | 93 | Ⅱ | 8 | 6 | 16 | 3.98 |
| 2 | 60 | M | 75 | 21.9 | 56 | 47 | N | Y | Y | 46 | Ⅲ | 20 | 9 | 19 | 63.50 |
| 3 | 41 | F | 80 | 28.0 | 26 | 181 | N | N | N | N | Ⅲ | 16 | 9 | 24 | 149.00 |
| 4 | 35 | F | 63 | 24.6 | 34 | 9 | Y | Y | Y | 9 | Ⅲ | 12 | 6 | 18 | 20.50 |
| 5 | 51 | M | 78 | 27.6 | 47 | 51 | N | Y | Y | 50 | Ⅳ | 26 | 16 | 23 | 98.00 |
| 6 | 36 | F | 50 | 20.3 | 32 | 46 | N | N | N | N | Ⅲ | 16 | 12 | 22 | 12.60 |
| 7 | 32 | F | 47 | 18.6 | 22 | 119 | N | N | Y | 38 | Ⅲ | 17 | 10 | 23 | 619.00 |
| 8 | 51 | F | 48 | 19.5 | 44 | 85 | Y | N | N | N | Ⅲ | 19 | 6 | 16 | 0.26 |
| 9 | 42 | F | 55 | 20.0 | 36 | 71 | Y | N | N | N | Ⅲ | 15 | 6 | 24 | 7.90 |
| 10 | 35 | F | 66 | 24.5 | 16 | 226 | N | N | N | N | Ⅳ | 23 | 7 | 17 | 21.40 |
| 11 | 50 | F | 40 | 15.6 | 41 | 98 | Y | N | N | N | Ⅲ | 12 | 8 | 22 | 1.22 |
| 12 | 29 | F | 51 | 19.7 | 20 | 107 | Y | N | N | N | Ⅳ | 19 | 13 | 14 | 5.16 |
| 13 | 32 | M | 66 | 22.1 | 13 | 230 | Y | N | N | N | Ⅲ | 16 | 6 | 20 | 116.00 |
| 14 | 40 | F | 83 | 32.0 | 23 | 197 | N | N | Y | 111 | Ⅲ | 23 | 7 | 17 | 6.06 |
| 15 | 39 | M | 70 | 24.2 | 37 | 23 | N | N | N | N | Ⅲ | 17 | 8 | 16 | 1.11 |
| 16 | 15 | F | 49 | 20.9 | 13 | 28 | Y | N | N | N | Ⅳ | 25 | 13 | 14 | 6.31 |
| 17 | 49 | F | 60 | 23.4 | 47 | 27 | N | N | N | N | Ⅲ | 14 | 8 | 22 | 17.10 |
| 18 | 48 | M | 96 | 30.3 | 19 | 340 | Y | N | N | N | Ⅳ | 20 | 17 | 23 | 119.00 |

AChR=acetylcholine receptor. BMI=Body Mass Index. F=female. M=male. MG-ADL=Myasthenia Gravis Activities of Daily Living. MGFA=Myasthenia Gravis Foundation of America. MG-QOL-15r=Myasthenia Gravis Quality of Life 15-revised. N=none. QMG=Quantitative Myasthenia Gravis. Y=yes. *The titer of AChR antibody was measured before lymphodepleting chemotherapy by radioimmunoassay at KingMed Diagnostic Laboratory in China.

## Table S2. Detailed treatment before CAR T-cell infusion

| No. | Previous treatment | | | | | | Ongoing treatment | | | | |
| --- | --- | --- | --- | --- | --- | --- | --- | --- | --- | --- | --- |
|  | Py | GC | NSISTs | Biologicals | IVIg | PLEX | Py | GC | NSISTs | Daily dose of Py (mg) | Daily dose of GC (mg) |
| 1 | Y | Y | TAC | RTX | Y | N | Y | Y | TAC | 180 | 10 |
| 2 | Y | Y | TAC, CTX | N | Y | Y | Y | Y | N | 240 | 20 |
| 3 | Y | Y | TAC, MMF | RTX, EFG | Y | N | Y | Y | TAC | 360 | 20 |
| 4 | Y | Y | TAC | EFG | Y | N | Y | Y | TAC | 240 | 27.5 |
| 5 | Y | Y | TAC, CTX | EFG, Ecu | Y | Y | Y | Y | CTX | 360 | 40 |
| 6 | Y | Y | TAC | EFG, Ecu | N | N | Y | Y | TAC | 240 | 15 |
| 7 | Y | Y | TAC | N | Y | N | Y | Y | TAC | 180 | 5 |
| 8 | Y | Y | MMF, TAC, CTX | N | N | N | N | Y | TAC | N | 25 |
| 9 | Y | Y | AZA, TAC | RTX | Y | N | Y | Y | N | 180 | 20 |
| 10 | Y | Y | TAC, CTX | N | Y | N | Y | Y | N | 120 | 20 |
| 11 | Y | Y | TAC | EFG, Ecu | Y | Y | Y | Y | TAC | 180 | 25 |
| 12 | Y | Y | TAC | N | Y | N | Y | Y | TAC | 480 | 5 |
| 13 | Y | Y | TAC | EFG, Ofatumumab | N | Y | Y | Y | N | 480 | 7.5 |
| 14 | Y | Y | AZA, TAC | EFG | N | N | Y | N | N | 120 | N |
| 15 | Y | Y | AZA, TAC | EFG | N | N | Y | Y | TAC | 180 | 20 |
| 16 | Y | Y | MMF, CTX | RTX, EFG | Y | N | Y | Y | N | 720 | 25 |
| 17 | Y | Y | MMF, TAC | EFG | N | Y | Y | Y | MMF | 180 | 27.5 |
| 18 | Y | Y | AZA, MMF | N | Y | N | Y | Y | AZA | 960 | 22.5 |

AZA=azathioprine. CAR=chimeric antigen receptor. CTX=cyclophosphamide. Ecu=Eculizumab. EFG=Efgartigimod. GC=glucocorticoid. IVIg=intravenous immunogloblin. RTX=rituximab. MMF=Mycophenolate Mofetil. N=none. NSIST=non-steroidal immunosuppressant. PLEX=plasma exchange. Py=pyridostigmine. Y=yes.

## Table S3. Percentage of BCMA/CD19 CAR positive T cells

| Patient number | Percentage of infused CD3+ cells expressing CAR-BCMA/CD19 (%) |
| --- | --- |
| Patient 1 | 58.3 |
| Patient 2 | 56.3 |
| Patient 3 | 75.0 |
| Patient 4 | 62.5 |
| Patient 5 | 50.0 |
| Patient 6 | 60.0 |
| Patient 7 | 52.5 |
| Patient 8 | 47.5 |
| Patient 9 | 55.6 |
| Patient 10 | 55.6 |
| Patient 11 | 66.7 |
| Patient 12 | 50.9 |
| Patient 13 | 55.6 |
| Patient 14 | 70.0 |
| Patient 15 | 46.0 |
| Patient 16 | 56.7 |
| Patient 17 | 37.7 |
| Patient 18 | 45.7 |

BCMA=B-cell maturation antigen. CAR=chimeric antigen receptor.

## Table S4. Summary of cytokine release syndrome

|  | Dose level 1 (n=6) | Dose level 2 (n=6) | Dose level 3 (n=6) | Total (n=18) |
| --- | --- | --- | --- | --- |
| Dose of CAR T cells | 1.0×10^6^/kg | 3.0×10^6^/kg | 5.0×10^6^/kg | － |
| Number of CRS, n (%) | | | | |
| Grade 1 | 1 (17%) | 5 (83%) | 1 (17%) | 7 (39%) |
| Grade ≥2 | 0 | 0 | 0 | 0 |
| Time to onset of CRS (day) | 26 | 9, 21, 8, 8, 11 | 9 | － |
| Duration of CRS (day) | 1 | 1, 2, 1, 1, 1 | 3 | － |
| Treated with dexamethasone | 0 | 1 (17%) | 0 | 1 (17%) |

CAR=chimeric antigen receptor. CRS=Cytokine release syndrome.

## Table S5. Summary of grade 3-4 hematological toxicities

|  | DL1 (n=6) | DL2 (n=6) | DL3 (n=6) | Total (n=18) |  | DL1 (n=6) | DL2 (n=6) | DL3 (n=5) | Total (n=17) |
| --- | --- | --- | --- | --- | --- | --- | --- | --- | --- |
|  | ≤28 days post-CAR T-cell infusion | | | |  | >28 days post-CAR T-cell infusion | | | |
| Leukopenia, n (%) | 1 (17%) | 1 (17%) | 1 (17%) | 3 (17%) | Leukopenia, n (%) | 1 (17%) | 0 | 0 | 1 (6%) |
| Neutropenia, n (%) | 0 | 0 | 0 | 0 | Neutropenia, n (%) | 2 (33%) | 0 | 1 (20%) | 3 (18%) |
| Lymphopenia, n (%) | 6 (100%) | 5 (83%) | 6 (100%) | 17 (94%) | Lymphopenia, n (%) | 0 | 0 | 0 | 0 |
| Anaemia, n (%) | 0 | 1 (17%) | 0 | 1 (6%) | Anaemia, n (%) | 0 | 0 | 0 | 0 |
| Thrombocytopenia, n (%) | 0 | 1 (17%) | 0 | 1 (6%) | Thrombocytopenia, n (%) | 0 | 0 | 0 | 0 |
| Febrile neutropenia, n (%) | 0 | 0 | 0 | 0 | Febrile neutropenia, n (%) | 0 | 0 | 0 | 0 |

CAR=chimeric antigen receptor. DL=dose level.

## Table S6. Changes in serum cytokine levels within 28 days after CAR T-cell infusion of all participants

| Cytokine concentration, pg/mL | Day 0 | | Day 3 | | Day 7 | | Day 10 | | Day 14 | | Day 21 | | Day 28 | |
| --- | --- | --- | --- | --- | --- | --- | --- | --- | --- | --- | --- | --- | --- | --- |
|  | CRS (n=7) | Without CRS (n=10) | CRS (n=7) | Without CRS (n=11) | CRS (n=7) | Without CRS (n=11) | CRS (n=7) | Without CRS (n=11) | CRS (n=7) | Without CRS (n=11) | CRS (n=7) | Without CRS (n=11) | CRS (n=7) | Without CRS (n=11) |
| IL-1β | 3.3 (2.7-3.7) | 3.1 (<2.5-6.7) | 3.3 (<2.5-4.3) | 3.1 (<2.5-3.5) | 3.6 (3.1-25.1) | <2.5 (<2.5-3.7) | 3.3 (<2.5-3.4) | 3.0 (<2.5-3.4) | 3.4 (<2.5-3.5) | 2.7 (<2.5-3.5) | 3.4 (<2.5-3.6) | <2.5 (<2.5-2.9) | 2.6 (<2.5-3.7) | <2.5 (<2.5-3.1) |
| IL-2 | 2.7 (<2.5-3.2) | 2.9 (<2.5-3.5) | 2.9 (<2.5-3.3) | 3.3 (2.6-5.1) | 3.1 (<2.5-3.4) | 2.8 (2.6-3.3) | 2.9 (2.8-2.9) | 2.6 (<2.5-3.3) | 3.1 (<2.5-3.4) | 3.1 (<2.5-3.5) | 2.9 (<2.5-3.6) | 3.0 (<2.5-3.7) | 3.0 (<2.5-3.3) | 3.1 (<2.5-3.6) |
| IL-5 | 2.7 (<2.5-3.6） | 3.2 (<2.5-4.0） | 2.9 (2.7-3.5) | 3.5 (<2.5-3.9) | 2.7 (<2.5-3.8) | 3.6 (<2.5-4.3) | 3.1 (2.8-3.7) | 2.6 (<2.5-4.0) | 2.6 (<2.5-2.8) | 3.7 (<2.5-4.2) | 3.0 (<2.5-3.9) | 3.1 (<2.5-4.3) | 3.1 (<2.5-3.8) | 2.7 (<2.5-4.2) |
| IL-6 | 9.6 (<2.5-21.5) | 3.0 (<2.5-8.7) | 10.1 (3.5-15.4) | 4.6 (<2.5-11.5) | 18.4 (2.8-22.2) | 5.2 (1.73-12.5) | 7.8 (4.2-13.6) | 4.0 (2.9-15.8) | 9.3 (2.97-10.6) | 3.6 (<2.5-7.2) | 30.0 (9.2-56.8) | 7.5 (3.7-13.4) | 7.0 (3.4-81.0) | 8.3 (2.7-18.9) |
| IL-8 | 11.0 (<2.5-28.5) | 2.8 (<2.5-5.7) | 6.6 (2.8-45.6) | 3.5 (3.0-12.5) | 3.6 (<2.5-16.4) | 6.5 (2.9-15.4) | 4.9 (3.5-8.2) | 6.7 (3.5-21.9) | 4.5 (<2.5-6.0) | 3.8 (3.2-8.6) | 2.7 (<2.5-4.0) | 3.2 (<2.5-7.8) | 2.5 (<2.5-9.8) | 3.4 (<2.5-6.7) |
| IL-10 | 4.8 (<2.5-5.7) | 5.4 (2.8-6.3) | 7.3 (3.8-10.4) | 7.6 (<2.5-16.1) | 22.0 (4.9-62.7) | 30.9 (<2.5-43.9) | 45.9 (19.2-71.1) | 15.3 (4.3-35.5) | 21.0 (3.7-30.1) | 7.0 (3.5-41.3) | 11.6 (5.5-15.0) | 10.8 (<2.5-43.9) | 5.7 (3.5-14.3) | 19.6 (2.7-41.3) |
| IL-12p70 | 3.0 (2.8-3.2) | 2.9 (2.3-3.1) | 2.8 (2.6-3.0) | 2.9 (2.2-3.0) | 2.9 (2.7-3.9) | 2.9 (<2.0-3.0) | 3.0 (2.8-3.2) | 2.8 (2.6-3.3) | 2.8 (2.5-3.7) | 2.8 (<2.0-3.0) | 2.9 (2.5-3.4) | 2.8 (2.6-3.1) | 2.8 (1.5-2.9) | 2.8 (1.5-2.9) |
| IFN-α | 3.0 (<2.5-3.7) | 2.6 (<2.5-4.2) | 3.0 (2.7-3.6) | 2.6 (<2.5-3.5) | 5.2 (<2.5-9.3) | 3.3 (<2.5-4.4) | 4.6 (<2.5-5.6) | 3.7 (2.7-5.4) | 4.2 (3.2-6.0) | 3.1 (<2.5-4.0) | 3.1 (<2.5-4.7) | 3.0 (2.4-3.7) | 3.4 (2.6-3.7) | 3.3 (3.0-3.6) |
| IFN-β | 3.1 (2.6-3.4) | 3.0 (2.6-3.6) | 2.9 (<2.5-3.0) | 3.2 (<2.5-4.1) | 4.7 (2.6-7.3) | 3.6 (<2.5-9.9) | 3.1 (<2.5-3.6) | 3.8 (<2.5-15.3) | 3.5 (2.6-4.14) | <2.5 (<2.5-5.1) | 3.4 (2.7-5.0) | 3.6 (<2.5-12.0) | 4.1 (3.3-9.0) | 5.3 (<2.5-11.7) |
| TNF-α | 2.6 (<2.5-5.5) | 2.7 (<2.5-3.7) | 2.9 (<2.5-4.2) | 2.9 (<2.5-3.5) | 2.8 (<2.5-3.3) | 3.5 (<2.5-5.5) | 3.1 (2.7-3.2) | 3.9 (3.0-4.7) | 3.0 (<2.5-4.7) | 3.0 (<2.5-4.4) | 3.0 (2.7-3.7) | 3.8 (<2.5-5.3) | 3.1 (<2.5-4.1) | 4.4 (<2.5-5.6) |

CAR=chimeric antigen receptor. CRS=Cytokine release syndrome. Interferon=IFN. Interleukin=IL. TNF=tumor necrosis factor.

## Table S7. Changes in daily dose of prednisone and pyridostigmine after CAR T-cell infusion.

| Patient number | Dose of prednisone (mg/day) | | | | Dose of pyridostigmine (mg/day) | | | |
| --- | --- | --- | --- | --- | --- | --- | --- | --- |
|  | D0 | D28 | D90 | D180 | D0 | D28 | D90 | D180 |
| Patient 1 | 10 | 2.5 | 0 | 0 | 180 | 180 | 0 | 0 |
| Patient 2 | 20 | 0 | 0 | 0 | 240 | 0 | 0 | 0 |
| Patient 3 | 20 | 20 | 0 | 0 | 360 | 360 | 0 | 0 |
| Patient 4 | 27.5 | 0 | 0 | 0 | 240 | 0 | 0 | 0 |
| Patient 5 | 40 | 20 | 0 | 0 | 360 | 180 | 0 | 0 |
| Patient 6 | 15 | 10 | 5 | 0 | 240 | 120 | 120 | 0 |
| Patient 7 | 5 | 5 | 0 | 0 | 180 | 180 | 60 | 0 |
| Patient 8 | 25 | 15 | 0 | 0 | 0 | 0 | 0 | 0 |
| Patient 9 | 20 | 12.5 | 5 | 0 | 180 | 180 | 60 | 0 |
| Patient 10 | 20 | 15 | 0 | 0 | 120 | 120 | 30 | 0 |
| Patient 11 | 5 | 15 | 0 | 0 | 180 | 60 | 0 | 0 |
| Patient 12 | 25 | 0 | 0 | 0 | 480 | 360 | 0 | 0 |
| Patient 13 | 7.5 | 5 | NA | NA | 480 | 480 | NA | NA |
| Patient 14 | 0 | 0 | 0 | 0 | 120 | 0 | 0 | 0 |
| Patient 15 | 20 | 20 | 0 | 0 | 180 | 180 | 0 | 0 |
| Patient 16 | 25 | 15 | 10 | 0 | 720 | 270 | 120 | 0 |
| Patient 17 | 27.5 | 20 | 12.5 | 5 | 180 | 180 | 120 | 0 |
| Patient 18 | 22.5 | 22.5 | 15 | 5 | 960 | 840 | 240 | 0 |

CAR=chimeric antigen receptor. D=day. NA=Not Available.

## Table S8. Changes in proportion of peripheral CD19+ B cells after CAR T-cell infusion.

| Patient number | Proportion of CD19+ B cells (%) | | | | | | | | | |
| --- | --- | --- | --- | --- | --- | --- | --- | --- | --- | --- |
|  | D-5 | D0 | D7 | D14 | D21 | D28 | D90 | D120 | D150 | D180 |
| Patient 1 | 1.02 | 1.32 | 0 | 0 | 0 | 0 | 0 | 0.11 | 0.11 | NA |
| Patient 2 | 11.28 | 8.19 | 0 | 0 | 0 | 0 | 0 | 0 | 0.09 | 0 |
| Patient 3 | 0 | 0 | 0 | 0 | 0 | 0 | 0 | 0 | 0 | 0 |
| Patient 4 | 12.71 | 4.1 | 0.2 | 0 | 0 | 0 | 1.69 | 11.23 | 19.71 | 22.25 |
| Patient 5 | 4.1 | 0.23 | 0 | 0 | 0 | 0 | 0 | 0 | 0 | 0 |
| Patient 6 | 14.74 | 1.33 | 0 | 0 | 0 | 0 | 0 | 0 | 0.26 | 2.27 |
| Patient 7 | 10.97 | 2.79 | 0 | 0 | 0 | 0 | 0.19 | NA | 2.48 | 9.02 |
| Patient 8 | 7.81 | 1.01 | 0 | 0 | 0 | 0 | 0 | 0.17 | 0 | 0 |
| Patient 9 | 0.12 | 0 | 0 | 0 | 0 | 0 | 0.64 | 1.94 | 5.44 | 15.46 |
| Patient 10 | 5.16 | 1.88 | 0 | 0 | 0 | 0 | 7.3 | 6.76 | 16.25 | 21 |
| Patient 11 | 4.29 | 5.93 | 0 | 0 | 0 | 0 | 0 | 0 | 0 | 0 |
| Patient 12 | 12.91 | 7.79 | 0 | 0 | 0 | 0 | 2.21 | 18.18 | 23.14 | 20.60 |
| Patient 13 | 0 | 0 | 0 | 0 | 0 | 0 | NA | NA | NA | NA |
| Patient 14 | 12.52 | 4.18 | 0 | 0 | 0 | 0 | 13.37 | 20.93 | 20.47 | 16.92 |
| Patient 15 | 14.33 | 2.69 | 0.46 | 0 | 0 | 0 | 0 | 0 | 0.79 | 5.39 |
| Patient 16 | 8.95 | 2.65 | 0 | 0 | 0.04 | 0.75 | 7.94 | 15.76 | 20.3 | 26.4 |
| Patient 17 | 12.92 | 5.03 | 0.18 | 0.14 | 0.42 | 0.11 | 0 | 0 | 0 | 0 |
| Patient 18 | 2.84 | 3.01 | 0.42 | 0.06 | 0 | 0.21 | 0.75 | 4.66 | 4.33 | 5.49 |

CAR=chimeric antigen receptor. D=day. NA=Not Available.

## Table S9. Changes in anti-AChR antibodies titer after CAR T-cell infusion.

| Patient number | Titer of anti-AChR antibodies (nmol/L)^*^ | | | |
| --- | --- | --- | --- | --- |
|  | D-5 | D28 | D90 | D180 |
| Patient 1 | 3.98 | 0.78 | <0.01 | <0.01 |
| Patient 2 | 63.50 | 13 | 0.49 | 0.11 |
| Patient 3 | 149.00 | 80.5 | 3.78 | 0.59 |
| Patient 4 | 20.50 | 10.5 | 2.07 | 1.7 |
| Patient 5 | 98.00 | 20.9 | 2.08 | 0.4 |
| Patient 6 | 12.60 | 2.94 | <0.01 | 0.33 |
| Patient 7 | 619.00 | 506 | 119 | 21.4 |
| Patient 8 | 0.26 | 0.03 | <0.01 | <0.01 |
| Patient 9 | 7.90 | 2.47 | 0.8 | 0.69 |
| Patient 10 | 21.40 | 8.8 | 3.15 | 2.79 |
| Patient 11 | 1.22 | 0.33 | <0.01 | <0.01 |
| Patient 12 | 5.16 | 67.1 | 3 | 0.1 |
| Patient 13 | 116.00 | NA | NA | NA |
| Patient 14 | 6.06 | 5.52 | 4.57 | 1.2 |
| Patient 15 | 1.11 | 0.65 | <0.01 | <0.01 |
| Patient 16 | 6.31 | 4.4 | 2.2 | 0.14 |
| Patient 17 | 17.10 | 4.15 | 0.15 | 0.24 |
| Patient 18 | 119.00 | 78 | 39.3 | 15.9 |

AChR=acetylcholine receptor. CAR=chimeric antigen receptor. D=day. NA=Not Available. ^*^The titer of AChR antibody was measured before lymphodepleting chemotherapy by radioimmunoassay at KingMed Diagnostic Laboratory in China. The reference threshold was 0.4 nmol/L.

# Supplement Figures

## Figure S1. Structure of the anti-BCMA/CD19 bispecific CAR T cells

The bispecific CAR was constructed by connecting an anti-BCMA scFv and anti-CD19 scFv into a second-generation format containing 4-1BB, as previously reported by our previous studies.^1,2^

| 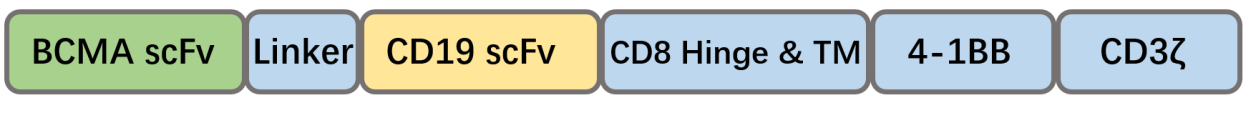 |
| --- |

BCMA=B-cell maturation antigen. CAR=chimeric antigen receptor.

## Figure S2. Changes in IL-6 and hs-CRP after CAR T-cell in all participants

| 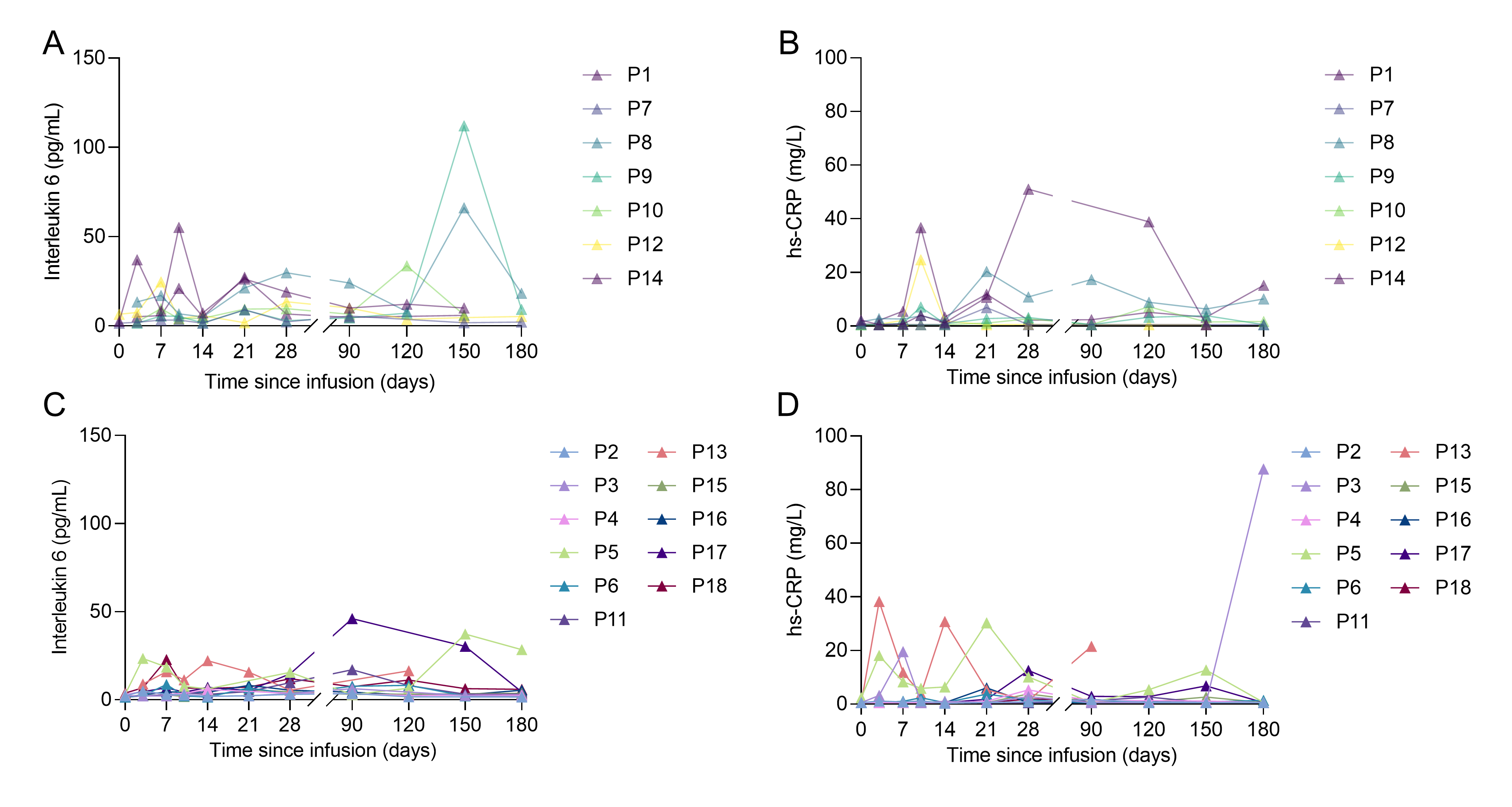 |
| --- |

CAR=chimeric antigen receptor. CRS=cytokine release syndrome. hs-CRP=high-sensitivity C-reactive protein. IL-6=interleukin 6.

## Figure S3. Changes in serum IgG, IgM, and IgA after CAR T-cell infusion in different dose

(A) IgG, (B) IgM, (C) IgA.

| 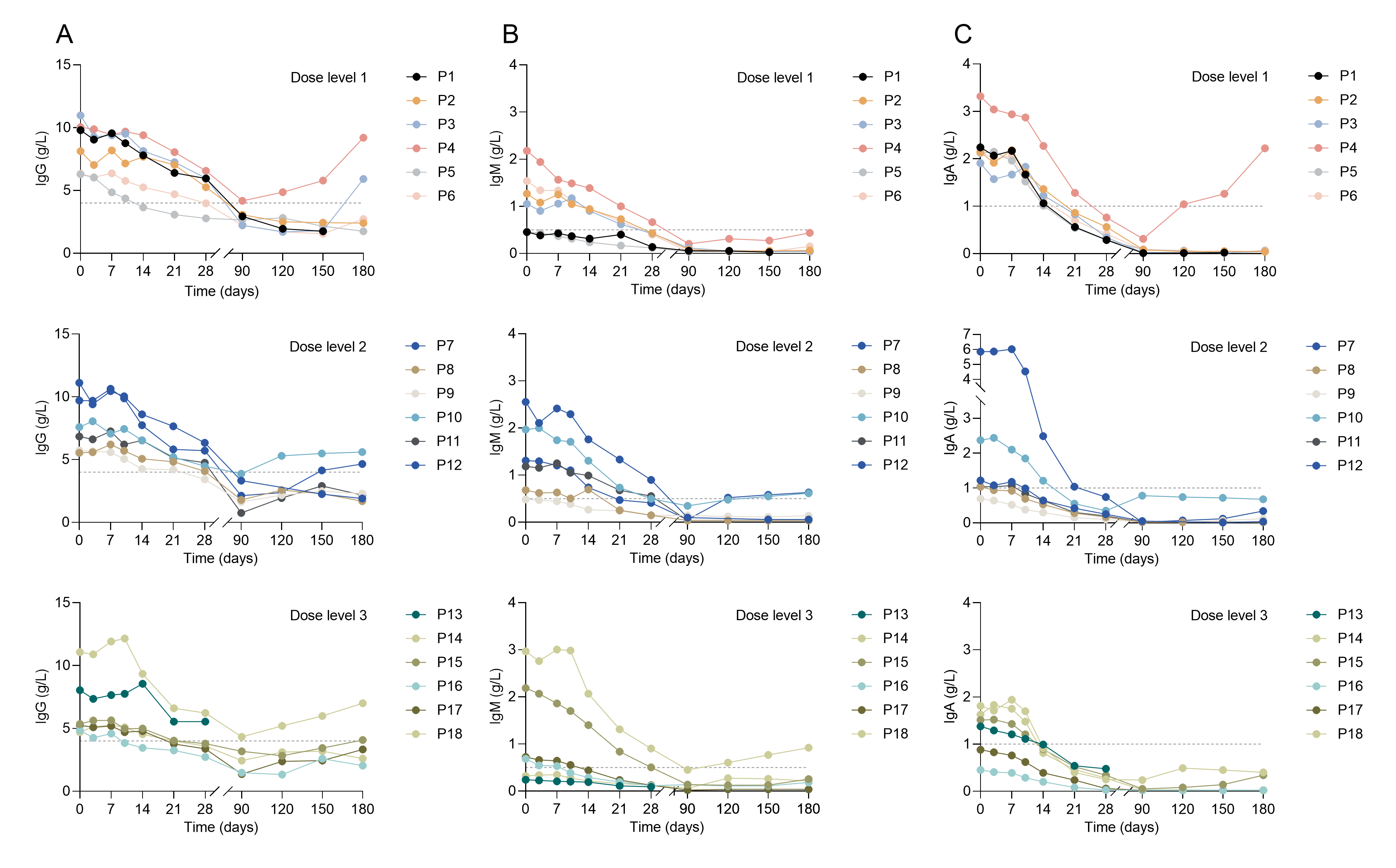 |
| --- |

CAR=chimeric antigen receptor. Ig=immunoglobulin.

## Figure S4. Amplification of anti-BCMA/CD19 bispecific CAR T cells

(A) Amplification of anti-BCMA/CD19 bispecific CAR T cells in patients with refractory myasthenia gravis within 28 days after CAR T-cell infusion. (B) Comparison of the peak copies of CAR T cells in different dose of CAR T-cell infusion.

| 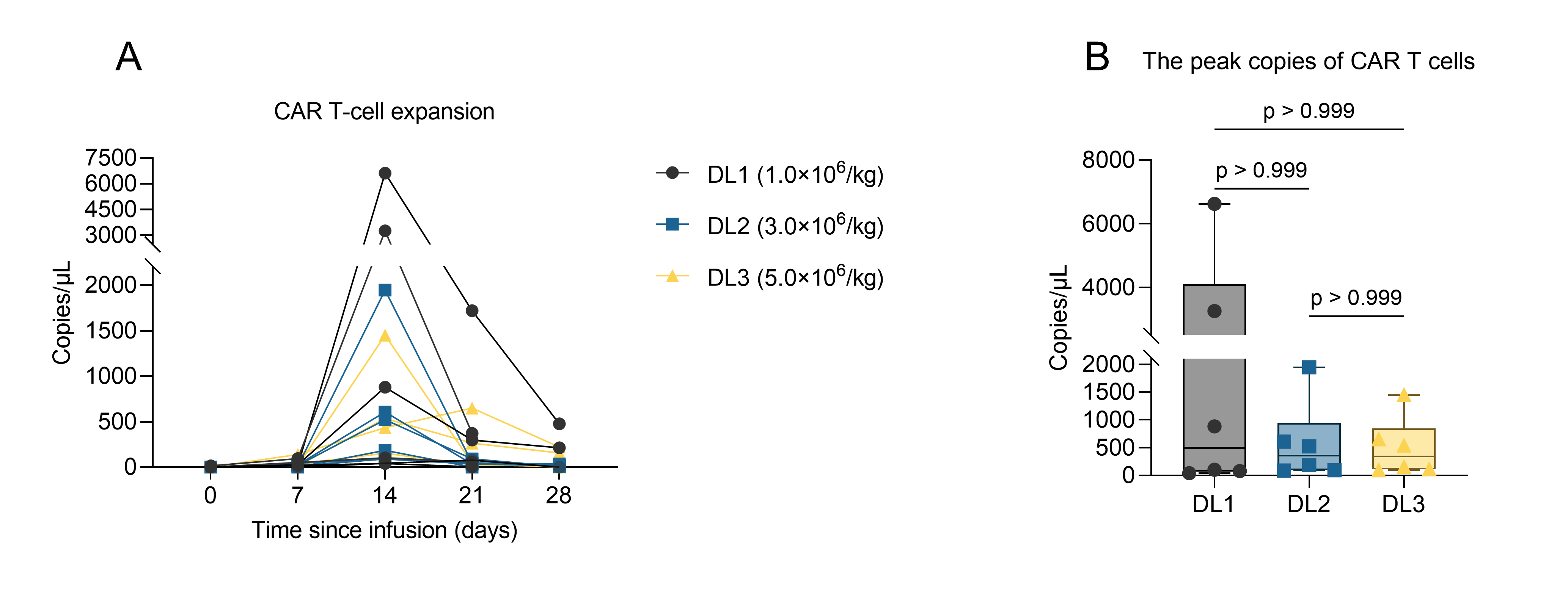 |
| --- |

BCMA=B-cell maturation antigen. CAR=chimeric antigen receptor.

## Figure S5. Changes in the proportions of CD3^+^ T, CD4^+^ T, CD8^+^ T, and NK cells among total lymphocytes after CAR T-cell infusion

(A) CD3^+^ T cells, (B) CD4^+^ T cells, (C) CD8^+^ T cells, (D) NK cells.

| 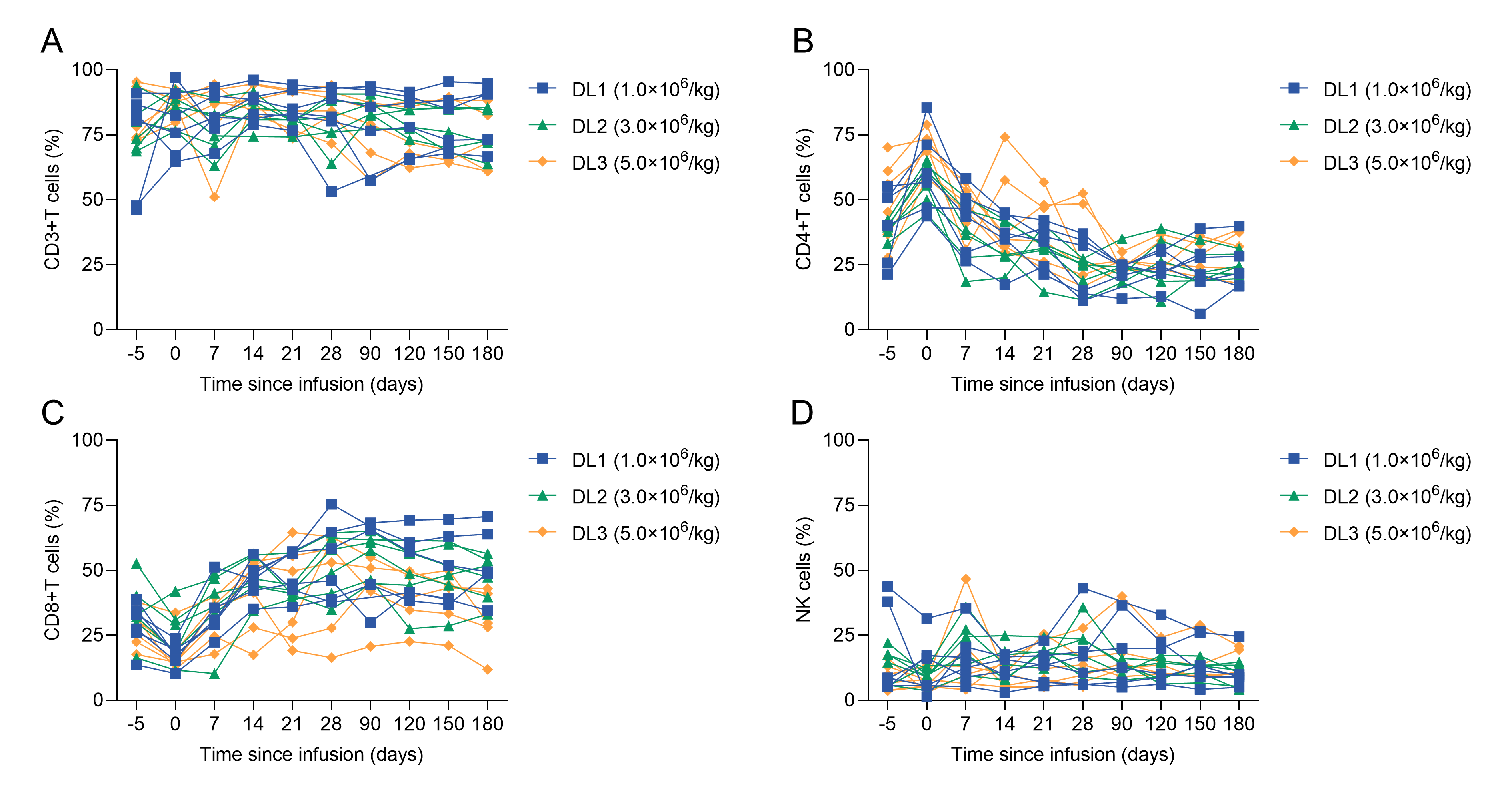 |
| --- |

CAR=chimeric antigen receptor. NK=natural killer.

## Figure S6. Changes in the proportion of participants with different MG-ADL score interval

| 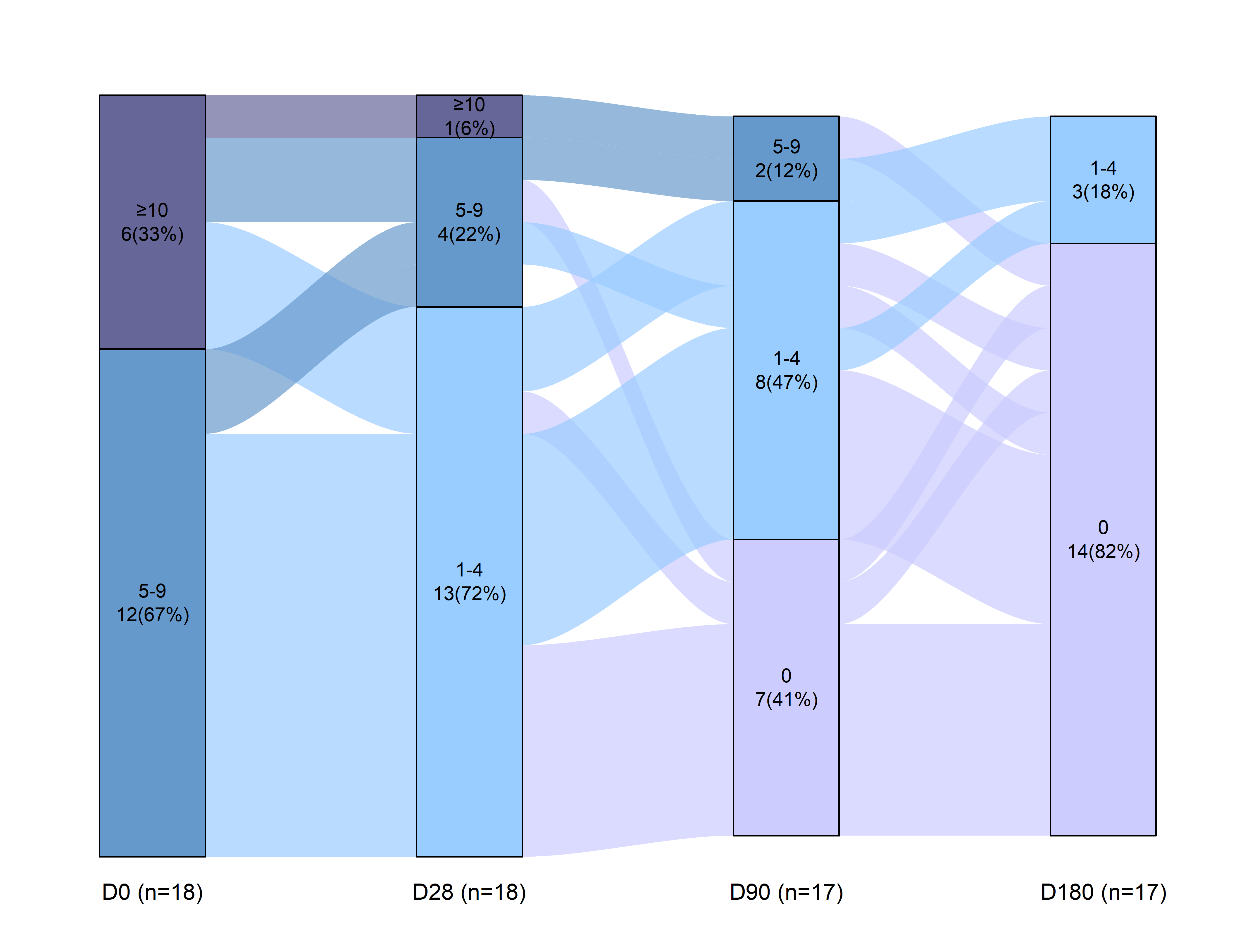 |
| --- |

MG-ADL=Myasthenia Gravis Activities of Daily Living. n=number of participants.

## Figure S7. Changes in the proportion of participants according to MGFA classification

| 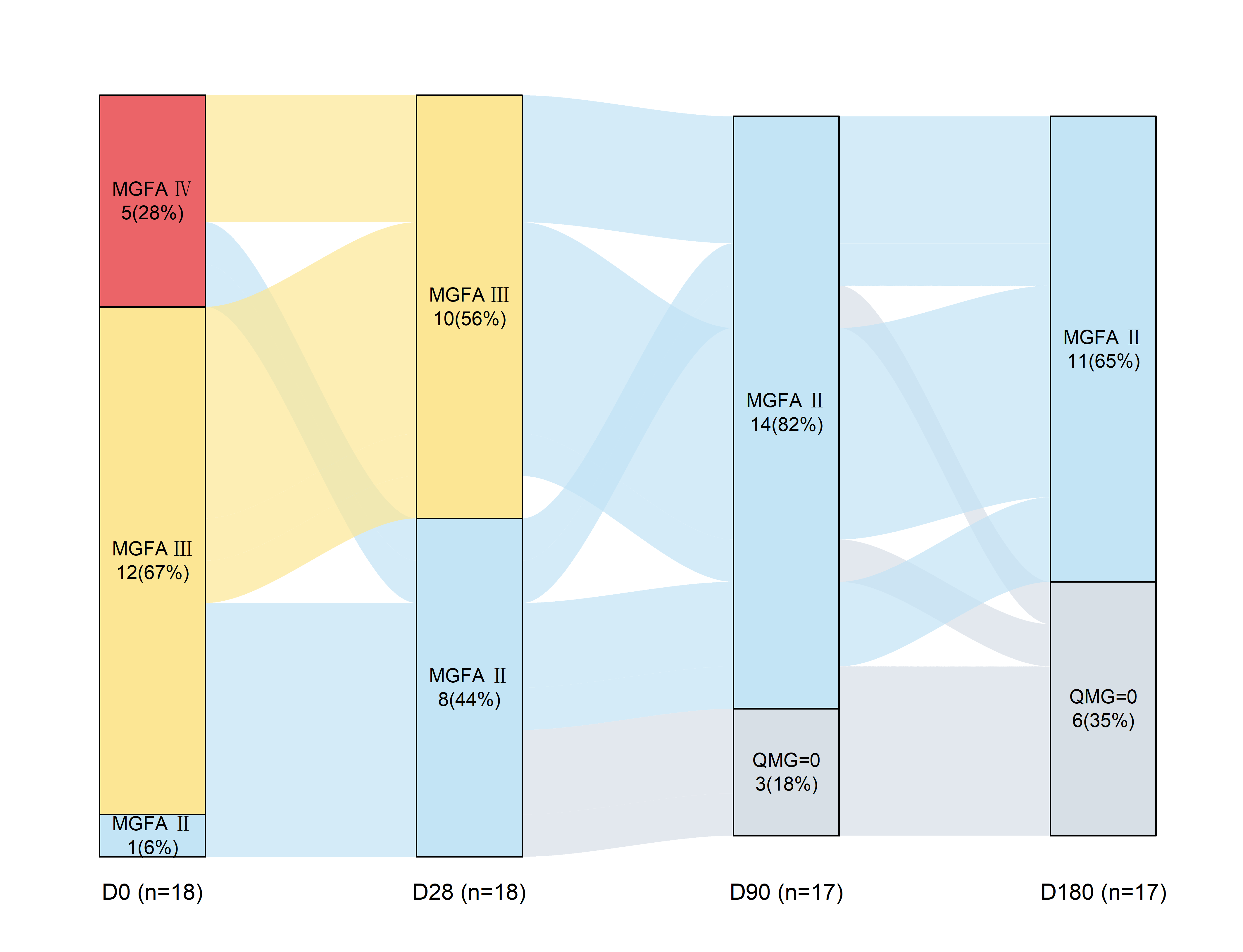 |
| --- |

MGFA=Myasthenia Gravis Foundation of America. n=number of participants. QMG=Quantitative Myasthenia Gravis.
